# The *Mycoplasma hyorhinis* p37 Protein Rapidly Induces Genes in Fibroblasts Associated with Inflammation and Cancer

**DOI:** 10.1371/journal.pone.0140753

**Published:** 2015-10-29

**Authors:** Amber Cathie Gomersall, Huy Anh Phan, Sylvana Iacuone, Song Feng Li, Roger W. Parish

**Affiliations:** Department of Animal, Plant and Soil Science, AgriBio, La Trobe University, Melbourne, Victoria, Australia; Institute of Biochemistry and Biotechnology, TAIWAN

## Abstract

The p37 protein at the surface of *Mycoplasma hyorhinis* cells forms part of a high-affinity transport system and has been found associated with animal and human cancers. Here we show in NIH3T3 fibroblasts, p37 rapidly induces the expression of genes implicated in inflammation and cancer progression. This gene activation was principally via the Tlr4 receptor. Activity was lost from p37 when the C-terminal 20 amino acids were removed or the four amino acids specific for the hydrogen bonding of thiamine pyrophosphate had been replaced by valine. Blocking the IL6 receptor or inhibiting STAT3 signalling resulted in increased p37-induced gene expression. Since cancer associated fibroblasts support growth, invasion and metastasis via their ability to regulate tumour-related inflammation, the rapid induction in fibroblasts of pro-inflammatory genes by p37 might be expected to influence cancer development.

## Introduction

The p37 protein was first discovered on the surface of mouse sarcoma FS9 cells [1]. Monoclonal antibodies directed against the p37 protein inhibited the invasive behaviour of the FS9 cells confronted by chicken heart fibroblasts [2]. The p37 protein was found to be from *Mycoplasma hyorhinis* and form part of a three protein high affinity transport system [3]. These proteins are highly similar to periplasmic binding high affinity transport systems of gram negative bacteria. The p37 N-terminus possesses the C-S-N amino acid sequence required for an N-terminal glyceride-cysteine lipid extension which inserts into the mycoplasmal membrane [4]. When *M*. *hyorhinis* was present, Rat-1 cells and FS9, L929 and NIH3T3 mouse fibroblasts all invaded chicken heart fibroblasts in the confronted explant assay [5]. If p37-specific monoclonal antibodies were added to the assay the invasive behaviour was inhibited.

The discovery of p37-induced cell invasivity suggested that *M*. *hyorhinis* infection might play a role in the development of cancer. *M*. *hyorhinis* infection has subsequently found to be associated with human and animal cancers including various carcinomas [6], as well as ovarian cancer and lymph node metastasis [7]. *M*. *hyorhinis* infection is correlated with metastasis and predicts poor survival of gastric cancer patients [8]. Fareed et al. analyzed the immune response of patients immunized intralymphatically with tumour cells and found patients exhibiting tumour regression had a measurable titre of antibodies against a 38 kDa protein [9]. Ilantzis et al. confirmed the protein to be p37 [10]. The p37 protein has been found associated with human gastric carcinomas and prostate tumours [11, 12]. Using an antibody targeting the N-terminus of p37, the protein was identified in gastric, colon, esophageal, lung, breast and glioma carcinomas as well as on circulating tumour cells from patients with hepatocellular carcinoma [6, 13].

Addition of p37 to human gastric carcinoma (AGS) cells increased migration in a transwell (Matrigel) assay [11]. Treatment of the prostate cancer lines PC-3 and DU145 with p37 also increased their invasivity through Matrigel [14]. Inclusion of a p37-specific monoclonal antibody inhibited this invasion. The level of metalloproteinase 2 (MMP2) increased in the media of p37-treated and *p37*-transfected AGS cells [11]. Goodison et al. suggest the increased invasivity may represent a greater migration rate following p37 treatment rather than increased capacity to degrade the Matrigel [15]. P37 treatment was also found to increase *tumor necrosis factor α* (*TNFα)* gene transcription and TNFα levels in the media of human peripheral blood mononuclear cells [16].

Various mycoplasmal infections have been associated with cancer and arthritis in animals and humans. For example, infection of 32D haematopoietic cells with *M*. *fermentans* or *M*. *penetrans* for 4–5 weeks induced malignant transformation and when injected into nude mice the cells rapidly formed tumours [17]. *M*. *hyorhinis*, *M*. *pneumoniae*, *M*. *hominis*, *M*. *fermentans*, *M*. *penetrans* and *M*. *arthritidas* have all been implicated in human arthritis [18–21]. *M*. *fermentans* produces acute arthritis in rabbits [21]. *M*. *hyorhinis* infection is also associated with polyserositis and arthritis of swine [22].

The aim of the work reported here was to identify genes whose expression is rapidly activated following p37 treatment of NIH3T3 fibroblasts *in vitro*. NIH3T3 cells were chosen as they are a standard fibroblast line that has proved valuable in the study of human disease including cancer.The membrane receptor(s) responsible for gene activation were also to be identified.

## Methods

### Plasmid construction

Plasmid construction was performed using standard restriction enzyme cloning. The *p37* gene was inserted into the *Bam*HI cut site of the pUC-derived pRSET A expression vector (Invitrogen; Cat# V351-20) (S1A Fig).

Truncated p37 was constructed using polymerase chain reaction (PCR) to introduce the *Bam*HI and *Nco*I restriction cut sites flanking the *p37* gene. The reverse primers (S1 Table) introduced an *Nco*I restriction cut site at several points which facilitated the production of DNA sequences that reduced the size of the p37 protein by 20, 60, 80 or 105 amino acids. The PCR products were digested with the *Bam*HI and *Nco*I restriction enzymes and ligated into the pUC-derived pRSET A expression vector (Invitrogen; Cat# V351-20) (S2 Fig).

### Site-directed Mutagenesis

Mutagenesis of the gene encoding p37 was performed using the MutaGene Phagemid *in vitro* mutagenesis kit (Bio-Rad; Cat# 170–3581). The oligonucleotides are supplied in S2 Table.

The four amino acids S255, F256, S257 and K258 in p37 were changed to valine using the QuikChange II XL Site-Directed Mutagenesis kit (Agilent Technologies; Cat#200521) and the primer design method developed by Zheng et al. [23]. Two polymerase chain reactions were used to carry out the mutations; the respective forward and reverse primers are listed in S3 Table and sequence analysis in S3 Fig. The optimum primer annealing temperature was established as 56.4°C.

### Protein expression and clarification

Expression of the p37 protein, truncated p37 proteins (p37-20, p37-60, p37-80 and p37-105) and the mutated p37 protein was completed in OneShot®BL21 (DE3) cells (Invitrogen; Cat#C6000-03). Cells were cultured in Luria-Bertani (LB) broth containing 100 μg ml^-1^ ampicillin and induced with IPTG (Isopropyl β-D-1-thiogalactopyranoside) to a final concentration of 1mM for 4 hours at 37°C with agitation. Induced cells were harvested and resuspended in Lysis buffer (50 mM NaH_2_PO_4_, 300 mM NaCl, 10 mM imidazole, 1 mg ml^-1^ Lysozyme, pH 8.0) containing a cOmplete, Mini Protease Inhibitor Cocktail Tablet (Roche; Cat#11836153001). Crude lysates were obtained by sonication (6 cycles, 30 second intervals) followed by agitation for 30 minutes at 4°C. The lysate was clarified by centrifugation at 12,000 g for 10 minutes at 4°C and pooled via filtration through a 25 μm filter.

### Arginine soak protein clarification

Higher concentrations of the p37 truncated peptides were located in the insoluble fraction of the *E*. *coli* lysate and so to increase yields of soluble p37 an arginine soak (argSOAK) method was employed. Truncated peptides were solubilised with a 1 M arginine soak prior to purification using the method described by Tsumoto et al. [24]. Native p37 was also purified using the 1 M arginine soak to ensure the method did not inactivate the protein.

### Protein purification

Protein purification was achieved using Profinity^TM^ IMAC Resin (BioRad; Cat#156–0123) with deviations from the manufacture’s protocol.

Two ml of the Profinity^TM^ IMAC Resin was added for every 25 ml of prepared cleared lysate. To allow binding of the protein the resin/lysate mixture was incubated for 1 hour at 4°C, with agitation. The mixture was then centrifuged for 1 minute at 3,000 g to pellet the resin. The resin was washed with 10ml Wash Buffer (50 mM NaH_2_PO_4_, 300 mM NaCl, 20 mM imidazole, pH 8.0) by agitation for 5 minutes at 4°C. The resin/wash mixture was centrifuged for 1 minute at 3,000 g to pellet resin. Protein absorbance readings at 280 nm (A_280_) were taken of the wash supernatants. The resin was repeatedly washed until the wash supernatants A_280_ was less than 0.01.

Elution was achieved by the addition of 7 ml Elution Buffer (50 mM NaH_2_PO_4_, 300 mM NaCl, 500 mM imidazole, pH 8.0) for every 2 ml of resin, with 1 hour incubation, agitation at 4°C. The mixture was centrifuged for 1 minute at 3,000 g to pellet resin and for every 7 ml, five 1 ml aliquots of the supernatant containing the eluted protein were collected. The eluted protein was stored at -80°C.

Protein concentrations were estimated following the Pierce® BCA Protein Assay Kit (ThermoScientific; Cat#23227) and the BCA program of the Eppendorf BioPhotometer 6131.

### SDS-PAGE, Coomassie Blue staining and WESTERN blotting

Protein samples were denatured by a 95°C heat treatment for 10 minutes and separated on 12% acrylamide separating gels with a 4% acrylamide stacking gel. The gels were constructed following the Mini-PROTEAN^®^ 3 Cell (BioRad; Cat# 165-3301/165-3302) manufacturer’s instructions. The Mini-PROTEAN 3 Cell Mini Tank (BioRad; Cat# 165–3302) was assembled according to the manufacturer’s instructions and the gels ran for 60 minutes, 200 volts at 4°C (Bio-RAD PowerPac^TM^ Basic power Supply).

To determine purification efficiency SDS-PAGE gels were stained with 0.1% Coomassie Blue Stain (0.1% Coomassie Blue R-250, 40% Methanol, 10% Acetic acid) overnight at room temperature with gentle agitation. The SDS-PAGE gels were fixed prior to Coomassie Blue staining with Fixing Solution (50% Methanol, 10% Acetic acid) at room temperature for 10 minutes with gentle agitation. Following Coomassie Blue staining gels were destained using a 40% Methanol, 10% Acetic acid destaining solution for 2 hours at room temperature with gentle agitation.

For protein identification, after separation on 12% acrylamide separating gels, proteins were transferred to polyvinylidene fluoride membrane following the BIO-RAD Mini Trans-Blot^®^ Electrophoretic protocol (Cat# 170-3930/170-3935). The BIO-RAD Mini Trans-Blot^®^ Electrophoretic system ran for 2 hours at 200 volts.

Membranes were blocked in 5% non-fat milk in Tris buffered saline with Tween-20 (TBST buffer: 137 mM NaCl, 20 mM Tris, 0.1% Tween-20), incubated again for 1 hour with the T7-Tag monoclonal antibody (Novagen; Cat# 69522) diluted 1:10,000 in 5% non-fat dried milk followed by a 1 hour incubation with goat anti-mouse IgG Horseradish Peroxide (HRP) conjugate (Invitrogen; Cat# G-21040) diluted 1:10,000 in 5% non-fat dried milk.

WESTERN blots were developed using the Alkaline Phosphatase Conjugate Substrate Kit (BioRad; Cat# 170–6432). The membrane was exposed to light for 10 minutes with agitation and washed with ddH_2_O to stop reaction before scanning.

The PageRuler Prestained Protein Ladder (Fermentas; Cat# SM0671) was used to analyse protein size.

### Microarray Analysis

Total RNA was extracted from NIH3T3 fibroblasts treated with 15 μg ml^-1^ purified p37 protein for 24 hours or non-treated NIH3T3 fibroblasts using the RNeasy® Mini Kit (Qiagen; Cat# 74104). Three biological replicates were taken of each treatment. Genomic contamination was screened by PCR and eliminated using Deoxyribonuclease I, Amplification Grade (Invitrogen; Cat#18068–015) per the manufacturer’s instruction. RNA integrity and quality was checked using the Agilent 2100 Bioanalyzer (Agilent Technologies, Santa Clara, California, USA).

RNA was amplified and cDNA synthesized according to instructions in the Genechip® 3’ IVT Express Kit User Manual (Affymetrix; Cat# P/N702646 Rev.8). Following biotin labelling of cDNA and fragmentation, samples were hybridized to Affymetrix Mouse Genome 430 2.0 Arrays, washed using a Genechip® Fluidics Station and scanned using the Genechip® Scanner 3000. Microarray data was processed using the Affymetrix® Expression Console™ Software 1.2 (Affymetrix; Cat# P/N 702387 Rev. 2) and CLC Genomics Workbench 4.7 (CLC bio, http://www.clcbio.com; Vat#DK28305087).

### RT^2^ Profiler™ PCR Array System

Total RNA was extracted from NIH3T3 fibroblasts which had been treated with p37, p37 and S31-201, S31-201 only or non-treated NIH3T3 fibroblasts using the RNeasy® Mini Kit (Qiagen; Cat# 74104). Three biological replicates were taken of each treatment. Genomic contamination was screened by PCR and eliminated using Deoxyribonuclease I, Amplification Grade (Invitrogen; Cat#18068–015) per the manufacturer’s instruction. RNA integrity and quality was checked using the Agilent 2100 Bioanalyzer (Agilent Technologies, Santa Clara, California, USA).

RNA Reverse Transcription and the RT^2^ Profiler^TM^ Inflammatory Response & Autoimmunity PCR arrays (SABioscience; Cat# PAMM-077A-12) were performed following the manufacture’s instruction (SABiosciences; Part#1022A). Strong positive correlations of the cycle threshold (Ct) values between the PCR array biological replicates of each treatment indicated reliable qPCR detection of gene expression (S4 Fig).

An ANOVA analysis was performed comparing gene Ct values of the treated samples to the untreated control.

### Quantitative PCR (qPCR)

RNA was extracted using the RNeasy® Mini Kit (Qiagen; Cat# 74104) from three biological replicates of treated and non-treated NIH3T3 fibroblasts. The RNA was DNAse-treated (Invitrogen; Cat# 18068–015) before complementary DNA conversion (SuperScript^TM^ III, Invitrogen; Cat# 18080–044). Quantitative amplication of cDNA was performed in triplicate of each biological replicate using iQ^TM^ SYBR^®^ Green Supermix (BioRad; Cat# 170–3884) and qPCR oligonucleotides (S4 Table) using an iCycler iQ^TM^ Real-Time PCR Detection System (BioRAD; 170–8740). The same amplification conditions were used for all primer sets; initial denaturing at 95°C for 3 minutes; amplification process cycled 40x through denaturation at 95°C for 10 seconds, primer annealing 60^°^C for 30 seconds and then an extension at 72°C for 30 seconds. Emitted fluorescence was measured during the cycled extension phase. A final extension of 95°C for 1 minute was completed before the dissociation curve. The dissociation curve began at 55°C with an increase of 0.5°C until the final temperature of 95°C was reached.

Negative controls were checked to eliminate contamination and only a single peak was accepted in the dissociation curve of any tested gene. All amplified products were sequenced to indicate specificity of the qPCR oligonucleotides.

### Fold Change

To normalise the different concentrations between samples all genes of interest (GOI) Ct values were subtracted from the average Ct values of the two endogenous reference genes *βactin* and *GAPDH* (*Glyceraldehyde 3-phosphate dehydrogenase*); ΔCt (Eq 1). All Ct values were obtained from three biological replicates and three technical replicates of each biological replicate (N = 9).

$$\Delta \text{Ct}=\;\bar{\text{Ct}}\;\text{GOI}-\bar{\text{Ct}}\;\text{reference genes}$$ Equation 1

The difference between the gene of interest ΔCt of a treated sample and a control sample was calculated; ΔΔCt (Eq 2). The amplification efficiency of the exponential change per cycle per gene (E) of each primer was calculated from the percent efficiency (E%); E (Eq 3). Percent efficiencies of 100 ±10% and R^2^≥0.985 were deemed acceptable. All primer pair efficiencies can be found in S4 Table.

$$\Delta \Delta \text{Ct}=\;{\Delta \text{Ct}}_{\text{Treated}}-{\Delta \text{Ct}}_{\text{Control}}$$ Equation 2

E=E%×0.01+1 Equation 3

The ΔΔCt was used with the respective gene primer E value to calculate the relative fold change of gene expression due to a treatment (Eq 4). A normalised ΔΔCt > 1 indicates upregulation and < 1 indicates downregulation.

$$\text{Fold Change}={\text{E}}^{-\Delta \Delta \text{Ct}}$$ Equation 4

### Error Bars

The standard deviation (*σ*) was calculated based on ΔΔCt (Eq 5). The standard error (SE) was calculated from the standard deviation (Eq 6) and the upper (Eq 7) and lower (Eq 8) error bars were calculated using the standard error, E-value and fold change. N, the number of the sample size = 9.

$$\sigma =\sqrt{\frac{\sum {\left(\Delta \Delta \text{Ct}-\bar{\Delta \Delta \text{Ct}}\right)}^{2}}{\text{N}}}$$ Equation 5

$$\text{SE}=\;\frac{\sigma }{\sqrt{\text{N}}}$$ Equation 6

$$\text{Upper}=\;\left[{\text{E}}^{-(\Delta \Delta \text{CT-SE})}\right]\;-\;\text{fold change}$$ Equation 7

$$\text{Lower}=\text{fold change}\;-\;\left[{\text{E}}^{-(\Delta \Delta \text{CT}+\text{SE})}\right]$$ Equation 8

Error bar values for all graphs are supplied in S5 Table.

### Analysis of Variance (ANOVA)

An ANOVA analysis was performed on all qPCR data comparing the normalized cycle threshold (ΔCt) of treated samples to the controls or treated control samples. All experiments consisted of three biological replicates and three technical replicates of each biological replicate (N = 9).

### Mammalian cells

Mouse embryonic (NIH3T3) fibroblasts established from NIH Swiss mouse embryos, were obtained from The Peter MacCallum Cancer Centre, East Melbourne.

### Cell culture conditions

NIH3T3 fibroblasts were grown in Dulbecco’s Modified Eagle’s Medium (DMEM). The media was supplemented with 10% fetal bovine serum (FBS), NaHCO_3_ and penicillin-streptomycin. Plasmocin^TM^ (Invivogen) was added to all culture media at a concentration of 5 μg ml^-1^. The cell line and media was tested for mycoplasma contamination using Mycoplasma primers described by Uphoff and Drexler [25]. PCR amplification conditions involved an initial denaturing at 95^°^C for 3 minutes and subsequent amplification process cycled 32x through denaturation at 95^°^C for 10 seconds, primer annealing 65^°^C for 30 seconds and an extension at 72^°^C for 30 seconds. A final extension of 95^°^C for 1 minute was completed. The cell line and all experiment cell media were found negative for Mycoplasma contamination.

All culture plates were incubated in a Water-Jacketed Incubator (Forma Scientific). The incubator was automatically regulated at 37^°^C with 5% CO_2_.

### Inhibitors

The following inhibitors were used: IL6R inhibitor LEAF^TM^ purified anti-mouse/rat CD126 monoclonal antibody (IL6R*i*) (Biolegend; Cat# 115809) (AB_2127939) at a final concentration of 0.1 μg ml^-1^. The final concentration was determined using RT-PCR which showed that concentrations of IL6R*i* ranging from 0.1 to 0.5 μg ml^-1^ inhibited p37-induced *serum amyloid A3* (*Saa3*) expression. The chemical probe STAT3 Inhibitor VI (S31-201) (Santa Cruz Biotechnology; Cat# sc-204304) was used at a final concentration of 100 μM [26]. The Viral Inhibitor Peptide of Tlr4 (VIPER) and the VIPER control peptide CP7 was employed at a final concentration of 0.5 μM (IMGENEX; Cat# IMG-2011set). The VIPER inhibition concentration 0.5 μM was determined by treating NIH3T3 cells with 1 μg ml^-1^ lipopolysaccharide (LPS) (InvivoGen; Cat# tlrl-3pelps) inhibiting with 0.25, 0.5, 0.75, 1, 5, 10 and 25 μM VIPER. The final concentration of 0.5 μM VIPER was found to reduce expression of *Saa3* from 12-fold to 5-fold. CP7 was also found to inhibit LPS-induced *Saa3* expression at higher concentrations however at 0.5 μM CP7, *Saa3* expression was not significantly inhibited.

### Cell treatment

NIH3T3 fibroblasts for treatment were passaged into the required number of tissue culture plates to allow for three biological replicates per treatment. All NIH3T3 lines originated from the same freeze down batch at passage 10; treatment occurred before passage 15. Prior to treatment, DMEM10%FCS medium was aspirated and cell cultures washed twice with 1x phosphate buffered saline (PBS; 137 mM NaCl, 2.7 mM KCl, 10 mM Na_2_HPO_4_.2H_2_O, 2 mM KH_2_PO_4_, pH 7.4), unless otherwise stated.

For p37 treatment the required concentration of purified p37 was added to the DMEM10%FCS medium covering the cells and the cultures incubated for the required amount of time. In the case of time trials, cell treatments were initiated at times that allowed for all treatment courses to be synchronised and ready for RNA extraction at T_0_.

NIH3T3 fibroblasts were treated with inhibitors before 24 hour treatment with or without p37. Pre-treatment incubation time varied depending on the inhibitor; 1 hour for IL6R*i*, 2 hours for VIPER and CP7 and 24 hours for S31-201.

Treatments were terminated by washing and lysis of cells for immediate RNA extraction. Cells were observed post-treatment for toxicity levels, if there was an observable toxic effect the experiment was terminated.

### Migration assays

Cell migration was stimulated in a monolayer by using an *in vitro* scratch wound assay. NIH3T3 fibroblasts were grown to a 100% confluent monolayer and scratched using a sterile pipette tip, forming a wound of approximately 300 μm in diameter. Cell debris was washed away with 1x PBS and DMEM10%FCS was added with 25 μg ml^-1^ p37 for treated NIH3T3 fibroblasts. Cultures that had not reached a confluent monolayer after 24 hour treatment and wounds that were greater or less than 300 μm were excluded from analysis. Images were captured at 0, 14, 19, 24 and 38 hours. Six images were captured per time point per plate. Triplicate plates were completed at each time point (N = 18). Rate of cell migration was expressed as surface area (μm^2^) covered by migrating cells divided by time (hours). To establish the area into which the cells had migrated at each time point, the area of the wound at each time point was subtracted from the initial area of the wound. ImageJ was used to determine the area of the wound.

## Results

### Gene expression profiling of p37 treated NIH3T3 fibroblasts

**Fig 1:**
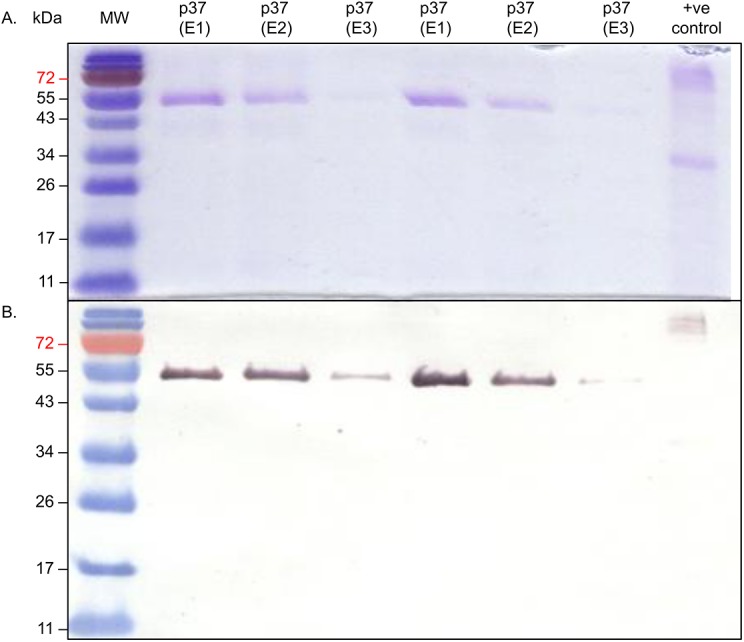
Purification of the p37 protein using Ni-affinity chromatography. Purified p37 was separated by 12% SDS-PAGE and stained with Coomassie blue **(A).** The purified protein was transferred to polyvinylidene fluoride membranes and probed with the T7-Tag monoclonal antibody and the goat anti-mouse IgG Horseradish Peroxide (HRP) conjugate (**B**). The molecular weight standards (MW) are in kilo Daltons (kDa) and indicated on the left of the figure. The purified p37 protein ran to the position of approximately 52 kDa. The p37 protein is predicted to be 43.5 kDa with an additional 8.5 kDa as a result of the 6x His Tag and Xpress Epitope of the pRSET A reading frame. The identity of the purified p37 protein was further confirmed using protein sequencing.

Recombinant *p37* gene expression was induced in *Escherichia coli* and the protein purified using Ni-affinity chromatography (Fig 1). Initially, the effect of the purified p37 on NIH3T3 fibroblast migration was determined. In a wound healing assay 25 μg ml^-1^ p37 treated fibroblasts exhibited increased migration rates (S5B Fig) and more rapid wound closure than the controls (S5A Fig). p37 did not affect the proliferation rate of NIH3T3 cells. There is one report of p37 treatment causing a slight increase in the proliferation of DU145 prostate cells (no data provided), however, PC3 prostate cells were unaffected (15).

NIH3T3 fibroblasts were incubated with 15 μg ml^-1^ p37 for 24 hours and a microarray analysis of the purified total RNA indicated expression of 537 genes significantly affected (p≤0.001); 288 of these genes were upregulated (fold change ≥ 3) (S6 Table). The gene ontology assignments for the 288 genes significantly upregulated are supplied in S6 Fig. The ten most strongly upregulated genes (9- to 64-fold) have all been reported to influence cancer progression and/or inflammation (see Discussion). We selected for analysis an additional eight genes (upregulated 3- to 9-fold) that also affect inflammation/cancer. The microarray data for the eighteen genes was validated using quantitative PCR (qPCR) (S7 Fig). Upregulation in response to p37 treatment was confirmed for fourteen of the genes. The fold changes remained relatively constant between the various (later) experiments. We subsequently used 25 μg ml^-1^ p37 and 24 hour treatments; fold changes were comparable between experiments. *Haptoglobin* (*Hp*) was an exception.

The microarray analysis identified 249 genes strongly downregulated (fold change ≥ 3) (S7 Table). Downregulation of five of these genes has been associated with tumour progression and the activation of acute phase protein (APP) genes (S8 Table).

### Effect of different p37 concentrations and treatment times

NIH3T3 fibroblasts were incubated with 0.5, 1, 5 and 25 μg ml^-1^ p37 for 24 hours. The lower p37 concentrations were less effective at stimulating gene expression although *Complement component 3* (*C3*) and *Lipocalin 2* (*Lcn2*) were still significantly activated by 5 μg ml^-1^ p37 (Table 1).

**Table 1:** Gene expression of NIH3T3 fibroblasts treated with different concentrations of purified p37 (0.5, 1, 5 and 25 μg ml^-1^) for 24 hours.

	p37			
	0.5 μg ml^-1^	1 μg ml^-1^	5 μg ml^-1^	25 μg ml^-1^
Angptl4	**1**	1	**3**	**20**
C3	**3**	**9**	**13**	**54**
Cast	**1**	**1**	1	**2**
Cp	1	1	2	**2**
Dcn	1	**3**	**2**	**11**
Fkbp5	1	1	1	1
Has2	1	1	**2**	**5**
Hp	**2**	2	**7**	**8**
IL6	**1**	1	1	**3**
Lcn2	**4**	**6**	**23**	**96**
LIF	1	1	**5**	**5**
Lum	1	1	1	**3**
Mmp9	1	**4**	**4**	**5**
Saa3	2	**2**	**7**	**197**
Thbs1	1	**2**	**1**	1
Tm4sf1	1	1	**1**	**3**
TNFαip6	**3**	2	2	**2**
Vcam1	1	2	1	2

Fold change (E^-ΔΔCt^) of mRNA expression levels of NIH3T3 fibroblasts treated with 0.5, 1, 5 or 25 μg ml^-1^ p37 for 24 hours. Significant differences between treated and untreated cells were calculated by ANOVA analysis (p-values ≤0.05 are shown in bold).

To determine changes in gene expression over time NIH3T3 fibroblasts were treated with 5 μg ml^-1^ p37 for 2, 4, 8, 12 and 24 hours. *Angiopoietin like-4* (*Angptl4*), *Serum Amyloid A3* (*Saa3*), *Vascular cell adhesion molecule 1* (*Vcam1*) and *Interleukin 6* (*IL6*) expression increased strongly during the first 4 hours of treatment but the activation had fallen to low levels or was absent at 24 hours (Table 2). The major increase in *Lcn2* and *C3* expression occurred between 12 and 24 hours. *Decorin* (*Dcn*) and *leukemia inhibitory factor* (*LIF*) expression increased during the first 4 hours, subsequently fell and then increased slightly again at 24 hours.

**Table 2:** Gene expression at different time points (2, 4, 8, 12 and 24 hours) following p37 (5 μg ml^-1^) addition to NIH3T3 fibroblasts.

	5 μg ml^-1^ p37					25 μg ml^-1^ p37
	2 hours	4 hours	8 hours	12 hours	24 hours	24 hours
Angptl4	**108**	**202**	**68**	**35**	**3**	**20**
Saa3	**46**	**80**	**66**	**15**	**7**	**197**
IL6	**42**	**98**	**7**	**4**	1	**3**
Vcam1	**17**	**21**	3	1	1	2
Dcn	**8**	**5**	**1**	1	**2**	**11**
LIF	**3**	**5**	1	1	**5**	**5**
Tm4sf1	**3**	**3**	**4**	2	1	**3**
Hp	2	2	**5**	**7**	**7**	**8**
Lcn2	**2**	**2**	**3**	**4**	**23**	**96**
Mmp9	**2**	**2**	**2**	**2**	**4**	**5**
Cp	**2**	**2**	**2**	**2**	2	2
Thbp1	**2**	2	1	2	1	1
C3	2	1	1	1	**13**	**54**
Has2	**2**	1	1	1	**2**	**5**
TNFαip6	1	1	1	**2**	2	**2**
Lum	1	1	**1**	**1**	1	**3**
Cast	**1**	**1**	**1**	1	1	**2**
Fkbp5	1	1	1	1	1	1

Fold change (E^-ΔΔCt^) of mRNA expression levels of NIH3T3 fibroblasts treated with 5 μg ml^-1^ p37 at 2, 4, 8, 12 or 24 hours. The 25 μg ml^-1^ p37 for 24 hours results are presented for comparison. Significant differences between treated and untreated cells were calculated by ANOVA analysis (p-values ≤0.05 are shown in bold).

Although some variability in fold change occurred when NIH3T3 fibroblasts were treated with 25 μg ml^-1^ p37 for 24 hours (Table 1 and later experiments), five genes *Angptl4*, *Saa3*, *Dcn*, *C3* and *Lcn2* were consistently activated more than 10-fold. Three genes *Hyaluronan synthase 2* (*Has2*), *Hp* and *LIF* by at least 5-fold.

### P37 activates gene expression via the Tlr4 receptor

**Fig 2:**
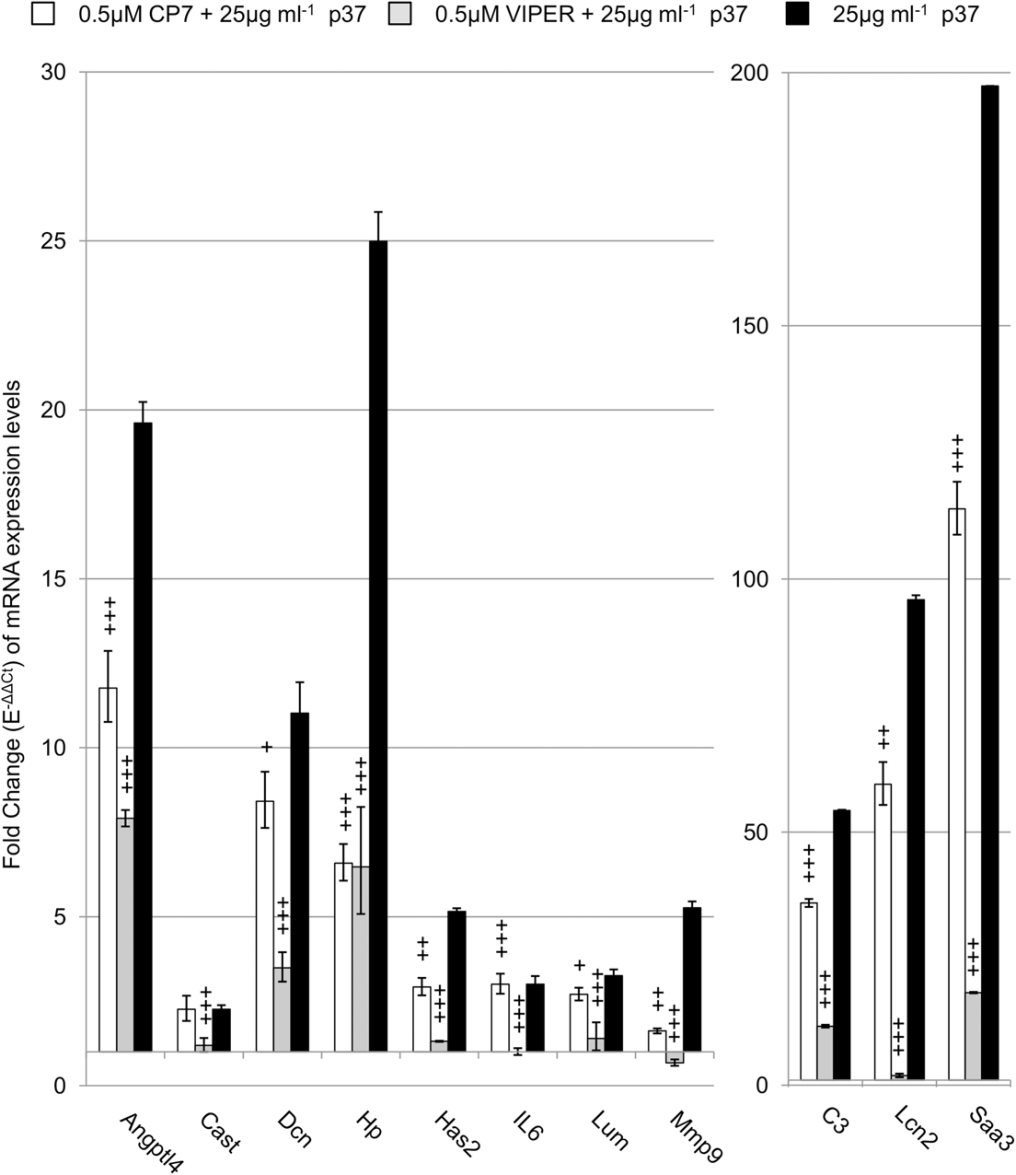
The effect of 0.5 μM VIPER and CP7 on p37-induced gene expression in NIH3T3 fibroblasts. Quantitative PCR (qPCR) analysis of NIH3T3 fibroblasts treated with 25 μg ml^-1^ p37 for 24 hours (black) or pre-treated for 2 hours with VIPER (grey) or the control peptide CP7 (white) prior to 25 μg ml^-1^ p37-treatment for 24 hours. Significant differences between CP7 or VIPER+p37 treatments and p37 treatment were calculated using ANOVA analysis (+p≤0.05, ++p≤0.01, +++p≤0.001).

The rapid increases in *Angptl4*, *LIF*, *Saa3*, *IL6* and *Vcam1* expression in p37 treated fibroblasts suggested the p37 protein is signalling via the toll-like receptor 4 (Tlr4). NIH3T3 fibroblasts possess Tlr4 since 1 μg ml^-1^ lipopolysaccharide (LPS) treatment for 6 hours resulted in a 28-fold increase in *IL6* expression in NIH3T3 fibroblasts [27]. We treated NIH3T3 fibroblasts with 1 μg ml^-1^ LPS for 24 hours and found a 2-fold and a 12-fold increase in *IL6* and *Saa3* expression, respectively (data not shown). The Viral Inhibitor Peptide of Tlr4 (VIPER) and its control peptide (CP7) [28] were used to determine whether p37 signals via Tlr4. NIH3T3 fibroblasts were incubated for 24 hours with p37 (25 μg ml^-1^) or pre-treated for 2 hours with the VIPER or CP7 peptides (0.5 μM) before the addition of p37. The effect of the peptides on the expression levels of the seven genes most strongly induced by p37 was determined (Fig 2). The p37-induced expression of all seven genes was significantly inhibited by VIPER. Although some CP7-induced inhibition occurred, in most cases this was significantly less than the inhibition caused by VIPER. Treatment of NIH3T3 fibroblasts with 0.5 μM VIPER or CP7 alone had no effect on the genes tested, with the exception of *Saa3* which was downregulated by 0.5 fold (S8A Fig).

### Truncation of p37 or mutating the TPP binding site inhibits gene activation

**Fig 3:**
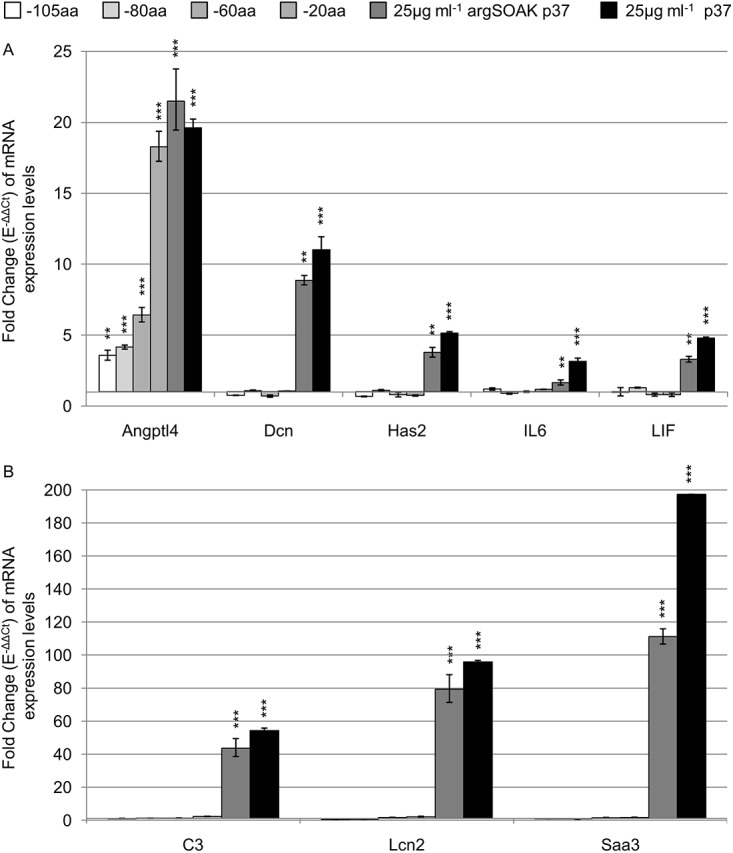
Effect of purified truncated p37 on gene expression. Quantitative PCR analysis of NIH3T3 fibroblasts treated with 25 μg ml^-1^ p37 excluding 20 amino acid (aa), 60aa, 80aa or 105aa (dark grey to white) from the C-terminus; for 24 hours. Arginine soak (argSOAK) purified p37 (darkest grey) slightly decreases p37-induced (black) gene expression in NIH3T3 fibroblasts. Significant differences between treated and untreated fibroblasts were calculated using ANOVA analysis (*p<0.05, **p<0.01, ***p<0.001).

Four truncated p37 peptides were prepared from which 20, 60, 80 or 105 amino acids had been removed from the C-terminus. Soluble peptides (25 μg ml^-1^) purified using the argSOAK method were used to treat NIH3T3 fibroblasts. The capacity of p37 to induce gene expression was lost when the C-terminal 20 amino acids were absent (Fig 3). The exception was *Angptl4* whose expression level induced by the 20 amino acid truncated peptide was similar to the full length p37 peptide. The expression levels of the other genes tested (seven are shown) were not significantly affected by the truncated p37 peptides.

**Fig 4:**
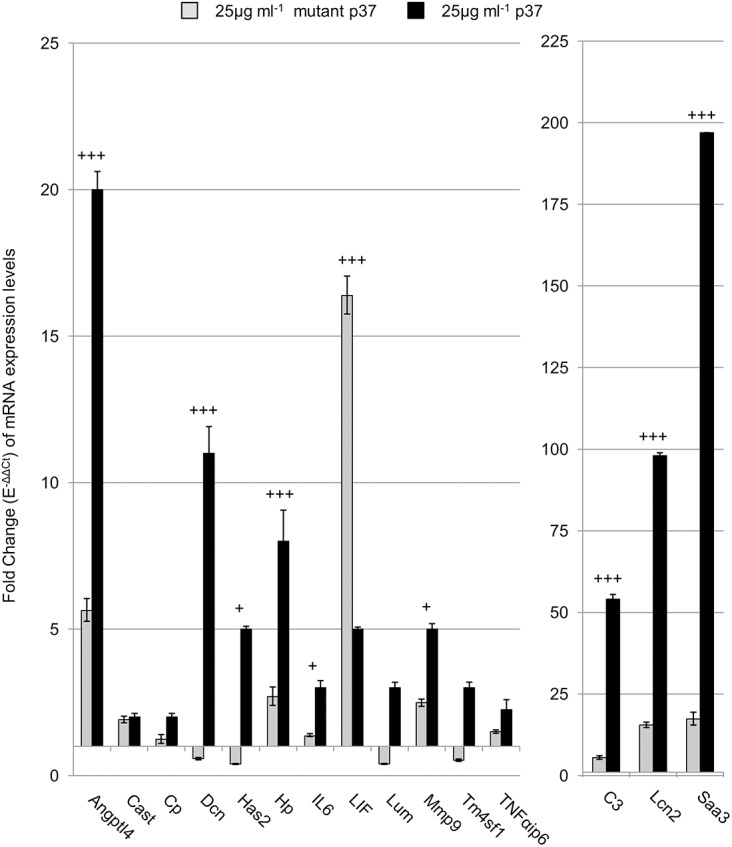
Mutant p37 affect on gene expression of NIH3T3 fibroblasts. Quantitative PCR analysis of NIH3T3 fibroblasts treated with either 25 μg ml^-1^ p37 with a mutated TPP binding site (grey) or native p37 (black) for 24 hours. Significant differences between mutant p37 and native p37 treatments were calculated using ANOVA analysis (+p<0.05, ++ p<0.01, +++p<0.001).

The crystalline structure of p37 has been defined to 1.9 Å resolution [29]. P37 is an alpha/beta class protein consisting of two domains separated by a cleft which modelling indicates binds thiamine pyrophosphate (TPP). The four amino acids S255, F256, S257 and K258 are specific to the hydrogen bonding of TPP. Site-directed mutagenesis was used to replace each of these four amino acids with valine. Mutant p37 upregulated *Angptl4*, *C3*, *Lcn2* and *Saa3* but to much lower levels than native p37 (Fig 4). However, the level of *LIF* expression was 3-fold higher with mutant p37 than with native p37.

### The effect of blocking STAT3 and IL6 signalling

Tlr4 activation results in an increase in *IL6* gene expression [27, 30] and IL6 activates signal transducer and activator of transcription 3 (STAT3) via the glycoprotein 130 (gp130) complex [31]. The IL6/STAT3 pathway activates the inflammatory response. To ascertain the extent to which increased IL6 levels and STAT3 signalling are responsible for the p37-induced gene expression, the effect of blocking IL6 and STAT3 signalling was determined.

**Fig 5:**
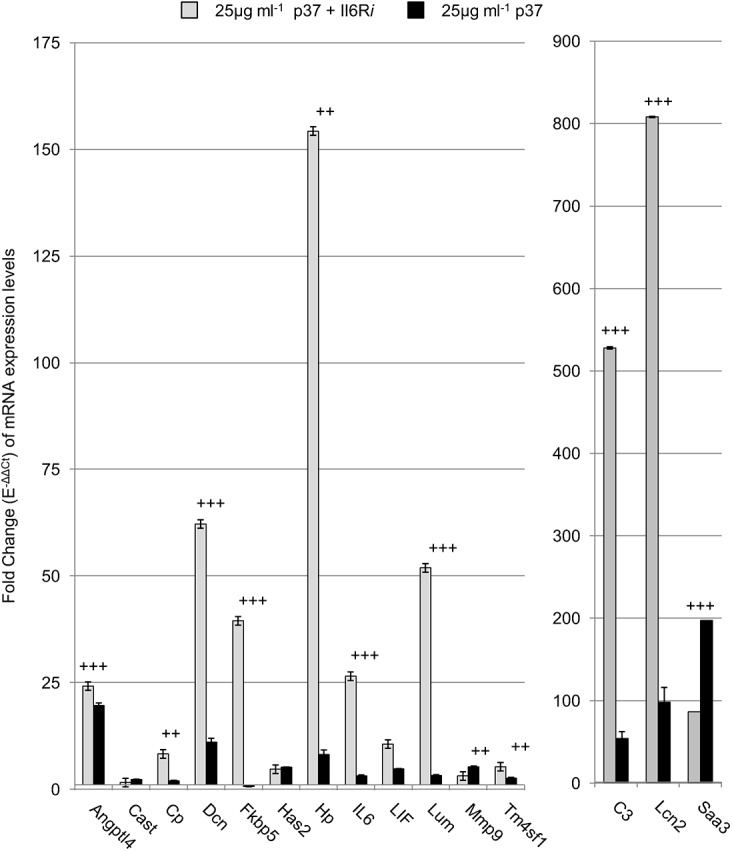
IL6R inhibition effect on p37-induced gene expression in NIH3T3 fibroblasts. Quantitative PCR analysis of NIH3T3 fibroblasts treated with 25 μg ml^-1^ p37 for 24 hours (black) or pre-treated with 0.1 μg ml^-1^ IL6R*i* for an hour prior to 25 μg ml^-1^ p37 treatment for 24 hours (grey). Significant differences between p37 + IL6R*i* treatment and p37 treatment was calculated using ANOVA analysis (+p<0.05, ++p<0.01, +++p<0.001).

NIH3T3 fibroblasts possess the IL6 receptor (IL6R) [32]. Cells were incubated with the IL6 receptor-α chain specific monoclonal antibody (IL6R*i*) which is directed against mouse/rat interleukin 6 receptor (IL6R) and its soluble counterpart (sIL6R). The antibody blocks IL6 binding to the gp130 receptor. When IL6R*i* (0.1 μg ml^-1^) was added with p37 (25 μg ml^-1^) to NIH3T3 fibroblasts, expression of ten of the fifteen genes tested was significantly higher than in p37 treated controls (Fig 5). The genes most strongly activated were *FK506 binding protein 5 (*Fkbp5; 56-fold greater than the p37-treated control), *Hp* (19-fold), *Lumican* (*Lum*; 16-fold), *C3* (10-fold), *IL6* (9-fold), *Lcn2* (8-fold) and *Dcn* (6-fold). *Saa3* expression was decreased by 60%. However, *IL6* and *Dcn* expression were increased 6-fold by IL6R*i* treatment alone (S8B Fig).

**Fig 6:**
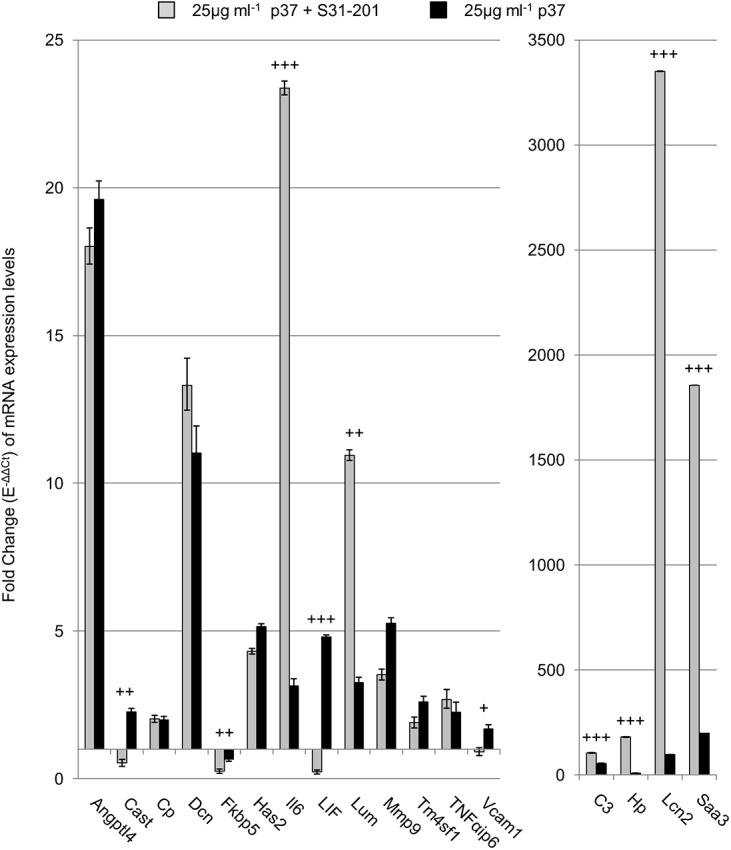
STAT3 inhibition effect on p37-induced gene expression in NIH3T3 fibroblasts. Quantitative PCR analysis of NIH3T3 fibroblasts treated with 25 μg ml^-1^ p37 for 24 hours (black) or 100μM of the STAT3 inhibitor S31-201 for 24 hours prior to 25 μg ml^-1^ p37 treatment for 24 hours (grey). Significant differences between S31-201 + p37 treatment and p37 treatment was calculated by ANOVA analysis (+p<0.05, ++p<0.01, +++p<0.001).

To ascertain the effects of blocking STAT3 activation on p37-induced gene expression we used the chemical probe S31-201. S31-201 binds to the Src homolog 2 (SH2) domain of STAT3, inhibiting STAT3 phosphorylation and dimerization [26]. Cells were incubated with S31-201 for 24 hours and then with 25 μg ml^-1^ p37 for a further 24 hours. S31-201 pre-treatment increased the p37-induced expression levels of *Lcn2* (35-fold greater than the p37-treated controls), *Hp* (22-fold), *Saa3* (9.5-fold), *IL6* (9.5-fold), *Lum* (3.5-fold) and *C3* (2-fold) (Fig 6). However, S31-201 inhibited p37-induced *LIF* expression by 95%. Treatment of NIH3T3 fibroblasts with 100 μM S31-201 alone slightly increased the expression (2 to 3-fold) of *Has2*, *IL6*, *Lcn2*, *Saa3* and *Hp* (S8C Fig).

Mouse inflammatory response and autoimmunity RT^2^ Profiler PCR arrays (SA Biosciences, Pam-077) were used to determine the response of 84 genes in cells treated with 25 μg ml^-1^ p37 (24 hours) plus or minus S31-201. No strong increases in expression were detected following p37 treatment, with the exception of *Chemokine (C-X-C motif) ligand 1* (10-fold) (Table 3). However, the combined (25 μg ml^-1^) p37 and S31-201 treatment significantly upregulated the expression of seven genes, including *IL6* and five chemokines.

**Table 3:** Genes identified in the inflammatory response and autoimmunity RT^2^ Profiler Array.

		Fold Change		
Gene Title	ID	25 μg ml^-1^ p37	S31-201	25 μg ml^-1^ p37 + S31-201
Chemokine (C-X-C motif) ligand 1	Cxcl1	**10**	3	**47**
Chemokine (C-C motif) ligand 2	Ccl2	4	3	**35**
Chemokine (C-X-C motif) ligand 5	Cxcl5	5	2	**33**
Chemokine (C-C motif) ligand 7	Ccl7	4	3	**33**
Interleukin 6	Il6	2	**5**	**25**
Chemokine (C-C motif) ligand 5	Ccl5	6	2	**13**
Interleukin 1 receptor, type I	Il1r1	1	**5**	**10**
Interleukin 10 receptor, beta	Il10rβ	2	**4**	**9**
Chemokine (C-X-C motif) ligand 10	Cxcl10	3	1	**6**
Lymphocyte antigen 96	Ly96	1	2	**6**
Interleukin 18	Il18	1	**4**	**6**
Receptor (TNFRSF)-interacting serine-threonine kinase 2	Ripk2	1	3	**4**
C-reactive protein, pentraxin-related	Crp	3	1	3
Nuclear factor of kappa light	Nfkb	1	1	**3**
Nuclear factor of activated T-cells, cytoplasmic, calcineurin-dependent 3	Nfatc3	1	2	**3**
Heat shock protein 90 alpha (cytosolic), class B member 1	Hsp90ab1	1	2	**3**
Hypoxanthine guanine phosphoribosyl transferase	Hprt	1	2	**3**
Toll-like receptor 1	TLR1	1	2	**3**
Toll-like receptor 2	TLR2	2	0.3	2
Histone deacetylase 4	Hdac4	2	2	2
Colony stimulating factor 1	Csf1	1	**3**	**2**
Interleukin 18 receptor accessory protein	Il18rap	1	**0.3**	**0.5**

Fold change (E^-ΔΔCt^) of mRNA expression levels of NIH3T3 fibroblasts treated with 100 μM S31-201 or 25 μg ml^-1^ p37 and 100 μM S31-201 over 24 hours. Significant differences between treated and untreated cells were calculated by ANOVA analysis (p-values ≤0.05 are shown in bold).

## Discussion

### P37 rapidly induces genes associated with inflammation and cancer

Treatment with the *M*. *hyorhinis* p37 protein rapidly (within 2 hours) induced expression of the *Angptl4*, *Dcn*, *IL6*, *LIF*, *Saa3* and *Vcam1* genes in NIH3T3 fibroblasts. *Dcn*, *IL6*, *LIF* and *Vcam1* are normally activated via the NF-κB (nuclear factor of kappa light polypeptide gene enhancer in B-cells) pathway. The proteins encoded by the six genes are secreted and have all been implicated in inflammation and cancer progression. IL6 is involved in inflammatory autoimmune diseases [33] and increased levels in a human ovarian cancer line results in anchorage independent growth, proliferation and invasion through Matrigel [34]. The anti-IL6R antibody tocilizumab is employed clinically to treat rheumatoid arthritis [35]. LIF has a role in the pathogenesis of arthritis [36].

Angptl4 is a positive acute phase protein which plays an important role in inflammation [37, 38] and cancer growth and metastasis [39]. Angptl4 released by tumour cells into the circulation increases lung capillary permeabilty and extravasation of cancer cells is facilitated. Two dimensional migration of various cell types *in vitro* is promoted by Angptl4 [40–43]. Human breast cancer cell invasion into a three dimensional Matrigel matrix is critically dependent on *Angptl4* expression [44]. Hence, the p37-induced *Angptl4* gene expression may at least in part be responsible for the increased migration rate and invasion of cells through Matrigel [11, 14]. *Angptl4* responded differently from the other genes induced by p37. Expression was still strongly induced by the 20 amino acid truncated p37 protein. P37-induction was not increased when STAT3 or IL6R were inhibited. Blocking Tlr4 signalling did partially block p37 induction of *Angptl4* but it appears an additional receptor(s) may be involved. *Angptl4* is normally activated by the glucocorticoid receptor and members of the peroxisome proliferated beta/gamma family.

Upregulation of *Saa3* occurs in rheumatoid arthritis and induces the transcription of matrix metalloproteinases [45]. Tlr4 acts as a Saa3 receptor in the pre-maturation phase of lung endothelial cells and macrophages, stimulating NF-κB and facilitating metastasis [46, 47]. Ectopic *Saa3* expression promotes metastasis in a breast cancer model [48]. SAA1 and SAA3 are effectors of the metastasis-promoting functions of the small calcium binding protein S100A4, providing a link between inflammation and tumour progression [48]. Saa3 induces toll-like receptor 2 (Tlr2) signalling in myeloid-derived suppressor cells resulting in increased TNFα secretion leading to STAT3 activation [49].

Vcam1 is a pro-inflammatory molecule [50] and facilitates breast cancer progression [51]. *Dcn* overexpression is required for efficient *in vitro* invasivity of a bladder tumour line [52].

The expression of a further four genes, namely *C3*, *Has2*, *Hp* and *Lcn2* was stimulated later (during 12–24 hours of p37 treatment). The proteins encoded by these genes are also secreted. Lcn2 promotes breast cancer progression [53] and is overexpressed in a variety of tumours [54, 55]. Hp and C3 are APPs. HAS2 promotes breast cancer cell invasion [56] and tumour progression in bowel cancer [57].

Inflammation is an important component of tumour progression [58]. Inflammatory cells are central to the tumour microenvironment as they facilitate proliferation, survival and migration. Cancer-associated fibroblasts (CAFs) support tumour growth, invasion and metastasis via their capacity to “orchestrate” tumour-related inflammation [59–61]. CAFs associated with human breast and ovarian tumours express high levels of *IL6*, a pro-inflammatory signal and component of the CAF pro-inflammatory gene signature [62]. NF-κB expression is upregulated in breast and ovarian tumours and is considered responsible for the activation of the pro-inflammatory genes. The secretion from fibroblasts of the proteins encoded by genes strongly induced by p37 is likely to influence inflammation and tumour progression.

Goodison et al. identified 38 and 23 genes whose expression was affected in the human prostate cell lines PC-3 and DU145, respectively, following p37 (25 μg ml^-1^) treatment for 4 hours [15]. Half of the genes were up-regulated. No fold changes were provided. The majority of these genes were not detected in our microarray, however, *IL6* expression was up-regulated in the PC-3 cells.

### Inhibiting IL-6R or STAT3 stimulates p37-induced gene activation

Tumourigenesis and metastasis are driven by the IL6/Janus kinase (JAK)/STAT3 feed-forward loop [63]. The *C3*, *Has2*, *Hp*, and *Lcn2* genes can be activated via IL6/gp130 or LIF/gp130/STAT3 pathways. However, inhibiting STAT3 or blocking IL6R resulted in stronger p37-induced gene activation. Others have also found an increased inflammatory response when STAT3 activity is inhibited. Lipopolysaccharide (LPS) activates Tlr4, eliciting a strong inflammatory response [64] and in STAT3 knockout (KO) mice LPS induced an exaggerated inflammatory response in multiple organs [65]. In IL6-stimulated mouse fibroblasts the genetic deletion of *STAT3* increased and prolonged STAT1 (signal transducer and activator of transcription 1) signalling and the upregulation of STAT1 and STAT3 target genes [66]. In IL6- and PH-Fib- (pulmonary hypertension adventitial fibroblast) stimulated macrophages, the blocking of STAT3 signalling gave rise to increased expression of STAT3-regulated genes associated with increased and prolonged STAT1 phosphorylation [67]. In the absence of STAT1, macrophages express higher levels of STAT3-regulated genes [68]. Thus, STAT1 and STAT3 are able to cross-regulate gene expression. Hence, one explanation for our results might be increased STAT1 signalling. Costa-Pereira et al. comment that such cross-regulation emphasizes the caution needed when using signalling inhibitors in clinical treatment [66].

Activated STAT3 can repress NF-κB target gene expression by binding to and sequestering NF-κB in the cytoplasm of human adenocarcinoma cells [69]. If activated STAT3 behaves similarly in NIH3T3 cells this could also explain the increase in p37-gene activation following STAT3 inhibition. Grabner et al. propose a re-evaluation of the therapeutic use of STAT3 inhibitors “for any inflammatory or fibrotic disease or cancer” [69].

Inhibition of IL6R with a monoclonal antibody also significantly increased p37-induced gene induction, suggesting IL6R involvement in a mechanism limiting the overexpression of those genes.

### P37 acts via the Toll-like 4 Receptor

Induction of gene expression by p37 was significantly reduced when Tlr4 signalling was inhibited, suggesting Tlr4 is the major cell surface receptor involved. Duan et al. have also recently reported that Tlr4 is involved in p37 signalling [69]. Co-immunoprecipitation and pull-down assays found p37-Tlr4 association in *M*. *hyorhinis* infected MGC803 human gastric cancer cells. The Tlr4 inhibitor CLE-095 blocked p37-activation of NF-κB and attenuated the EMT phenotype and migration of *M*. *hyorhinis* infected cells. An inhibitor of NF-κB signalling prevented *M*. *hyorhinis* and p37-induced migration of AGS and MGC803 cells. However, in AGS gastric cancer cells signalling appears to be via a different pathway. Tyr23 phosphorylation is associated with the cell surface translocation of annexin A2 (ANXA2) and both phosphorylation and translocation are enhanced by *M*. *hyorhinis* infection of AGS cells [8]. EGFR forms a complex with p37 and ANXA2 at the cell surface and the authors suggest that subsequent NF-κB pathway activation mediates *M*. *hyorhinis* driven cell migration. The mechanism by which intracellular ANXA2 phosphorylation may be affected by *M*. *hyorhinis* infection is not known. In *M*. *hyorhinis*-infected AGS cells expression of five of the six NF-κB target genes tested, namely *IκBα (nuclear factor of kappa light polypeptide gene enhancer in B-cells inhibitor alpha)*, *COX2 (Prostaglandin G/H synthase 2)*, *MAP1 (mannan-binding lectin serine peptidase 1)*, *PRDM1 (PR domain containing 1*, *with ZNF domain)* and *MMP1 (matrix metallopeptidase 1)* was increased by 2- to 8-fold [70].

The 20 amino acid C-terminus of p37 is exposed at the base of the two p37 domains (I and II) [29]. Based on the PyMOL schematic of p37 removal of the 20 amino acids should not affect the structural conformation of the remaining p37 tertiary structure. However, the loss of 20 amino acids from the C-terminus of p37 greatly reduced its capacity to induce gene expression. The exception was *Angptl4*, suggesting a receptor(s) in addition to Tlr4 may be involved. However, whether full length p37 also interacts with an additional receptor(s) is unknown. *Angptl4* also responded differently to other genes in that its p37-induced expression was not increased in the presence of IL6R or STAT3 inhibitors.

P37 is the substrate binding domain of an ABC transporter and thought to be anchored on the *M*. *hyorhinis* surface via an N-terminal glyceride-lipid extension [4]. Hence, the C-terminus would be exposed for contact with the mammalian cell Tlr4 receptor. Free p37 may be also present *in vivo*. Nothing is known about the stability of the anchored or free p37 *in vivo* or its rate of turnover on the *M*. *hyorhinis* surface.

The N-terminal region of recombinant p37 binds to ANXA2 and the p37-23 peptide blocks *M*. *hyorhinis* infection of MGC803 and AGS human gastric cancer cells [8]. Since p37 conformation is unlikely to change following removal of the C-terminal 20 amino acids [29], the N-terminal region would be expected to still be available for binding to a receptor(s), suggesting it is not involved in the gene activation described here.

When the four amino acids specific to the hydrogen bonding of TPP were mutated, the modified p37 failed to induce expression of the genes being studied with exception of *LIF* (3-fold increase). Hence, TPP appears to be required for p37 activity, presumably because the conformation of the protein changes in its absence.

Mycoplasma interacts with host cells in various ways including adherence, invasion and fusion. Mycoplasma infected tumour cells release exosomes which have surface-associated proteins and lipoproteins with potential pro-inflammatory properties [71]. Such exosomes would provide an additional means for exposing cells to p37.

In fibroblasts the rapid p37-induction of genes associated with inflammation and cancer suggests a mechanism by which *M*. *hyorhinis* infection could influence the development of arthritis and cancer in animals and humans. Whether p37 orthologues from other Mycoplasma species act in a similar way will be of interest. NIH3T3 cells were chosen because they have proved valuable for the study of human disease including cancer. They also provide a good model for evaluating the effects of p37 on gene expression in CAFs. The effects of p37 on gene expression in human fibroblasts would also be of interest although a number of lines would probably need to be studied as functional differences exist between fibroblasts from different organs.

## Supporting Information

S1 Fig Sequence alignment of *p37* cloned into the pRSET A vector. The *p37* gene, excluding the signal sequence, was cloned into the *Bam*HI cut site (green) of pRSET A (**A**). TGA codons mutated to TGG for tryptophan (W) expression in *E*. *coli* are indicated in blue. An extra base pair ‘g’ (yellow) was inserted to ensure *p37* was in frame for correct protein synthesis (**B**). Basic sequence alignment and analysis was performed utilising the program CLC Sequence Viewer 6 (Version 6.8.1).
(TIF)

S2 Fig Sequence analysis of the four truncated p37 constructs (p37-20, -60, -80 and -105). The locations of the forward and reverse primers are highlighted in blue. Basic sequence alignments and analysis was performed utilising the program CLC Sequence Viewer 6 (Version 6.8.1).
(TIF)

S3 Fig Sequence alignment of mutant *p37* in pRSET A. Several mutations (blue) were introduced into *p37* using site-directed mutagenesis. Site-directed mutagenesis was completed using two sets of primers (F1/R1) and (F2/R2) (S5 Table). Basic sequence alignments and analysis was performed utilising the program CLC Sequence Viewer 6 (Version 6.8.1).
(TIF)

S4 Fig Strong correlation between biological replicates of the inflammatory response and autoimmunity RT^2^ Profiler Array. Correlation plots of 96 gene Ct values between the triplicate Profiler array biological replicates (BioRep1, 2 and 3) for S31-201 treated NIH3T3 cells (**A**) and 25 μg ml^-1^ p37 treated NIH3T3 cells, pre-treated with S31-201 (**B**) (N = 96). Strong Pearson correlation coefficients (*r*), determined by the linear regression, are indicated.
(TIF)

S5 Fig (A) p37 enhances wound healing of NIH3T3 fibroblasts. NIH3T3 fibroblasts were treated with 25 μg ml^-1^ p37 for 24 hours reaching a confluent monolayer. The confluent monolayer was wounded with a sterile pipette tip. Three biological replicates and three technical replicates were analysed for each time point (N = 18) with the best representatives shown. ImageJ was used to determine the wound location (area absent of fibroblasts) which has been blackened out to better visualise cell migration. Scale bar 300 μm. (B) Rate of NIH3T3 fibroblast migration (μm^2^ hr^-1^) increases due to p37 treatment. Confluent monolayers of NIH3T3 fibroblasts were wounded and treated with 25 μg ml^-1^ p37. The rate of cell migration was calculated by the surface area (μm^2^) covered by migrating cells (closing the wound) divided by time (hours). The data represents the mean ± S.E. of three separate biological replicates and three technical replicates (N = 18). (TIF)

S6 Fig Gene Ontology (GO) assignments of significantly upregulated (p≤0.001, fold change ≥ 3) genes identified in the microarray. Cellular components (**a**), molecular processes (**b**) and biological processes (**c**) regulated by the genes significantly upregulated by p37, assessed by Gene Ontology (GO). GO classifications are extracted from the Mouse Genome Informatics Database. Note: An individual protein can be associated with more than one GOterm.
(TIF)

S7 Fig Microarray validation by qPCR. NIH3T3 fibroblasts were treated with 15 μg ml^-1^ of purified p37 for 24 hours. Quantitative PCR (qPCR) was used to validate p37-induced expression of 18 genes (p ≤ 0.001, fold change ≥ 3) identified in the microarray analysis using Affymetrix Mouse Genome 430 2.0 Arrays. Black bars represent Microarray mRNA levels expressed as absolute fold change (treated vs. untreated); p-values ≤ 0.001. Gray bars represent qPCR mRNA levels expressed as fold change (E^-ΔΔCt^) relative to untreated controls and normalized to the reference genes *GAPDH* and *βactin*. Significant differences between treated and untreated cells were calculated by ANOVA analysis (*p<0.05, **p<0.01, ***p<0.001).
(TIF)

S8 Fig Inhibitor effects on NIH3T3 fibroblasts. qPCR analysis of NIH3T3 fibroblasts treated with 0.5 μM VIPER or CP7 for 26 hours (**A**), 0.1 μg ml^-1^ IL6R antibody inhibitor (IL6R*i*) for 25 hours (**B**) or 100 μM S31-201 for 48 hours (**C**). Significant differences between treated and untreated cells were calculated by ANOVA analysis (*p<0.05, **p<0.01, ***p<0.001).
(TIF)

S1 Table Oligonucleotide sequences for amplification of C-terminal truncated mutants by PCR. *Nco*I restriction enzyme cut site in the reverse primers and *Bam*HI in the forward primers are indicated by underline.
(TIF)

S2 Table Sequences of the TGA to TGG (underline) mutagenic oligonucleotides. (TIF)

S3 Table Primer sequences for site-directed mutagenesis. The bold base pairs are those introducing point mutations to the *p37* gene. Oligonucleotides Forward 1 and Reverse 1 were used in the first PCR and Forward 2 and Reverse 2 were used in the second PCR.
(TIF)

S4 Table Quantitative PCR gene oligonucleotides. (TIF)

S5 Table The qPCR analysis standard errors of various NIH3T3 fibroblast treatments. (TIF)

S6 Table Dataset obtained from the microarray analysis of 24 hours, 15 μg ml^-1^ p37 treated NIH3T3 fibroblasts. The dataset consists of 288 genes significantly upregulated by ≥ 3 fold with a p-value of ≤ 0.001. Genes chosen for further study are indicated in bold.
(PDF)

S7 Table Dataset obtained from the microarray analysis of 24 hours, 15 μg ml^-1^ p37 treated NIH3T3 fibroblasts. The dataset consists of 249 genes significantly downregulated by ≥ 3 fold with a p-value of ≤ 0.001.
(PDF)

S8 Table Genes among the most downregulated by p37 whose downregulation may influence cancer progression. (TIF)
