# Supplementary figures and images for: The Mycoplasma hyorhinis p37 Protein Rapidly Induces Genes in Fibroblasts Associated with Inflammation and Cancer
Source: PLoS One. 2015 Oct 29;10(10):e0140753. doi: 10.1371/journal.pone.0140753 (PMC4626034; doi:10.1371/journal.pone.0140753)

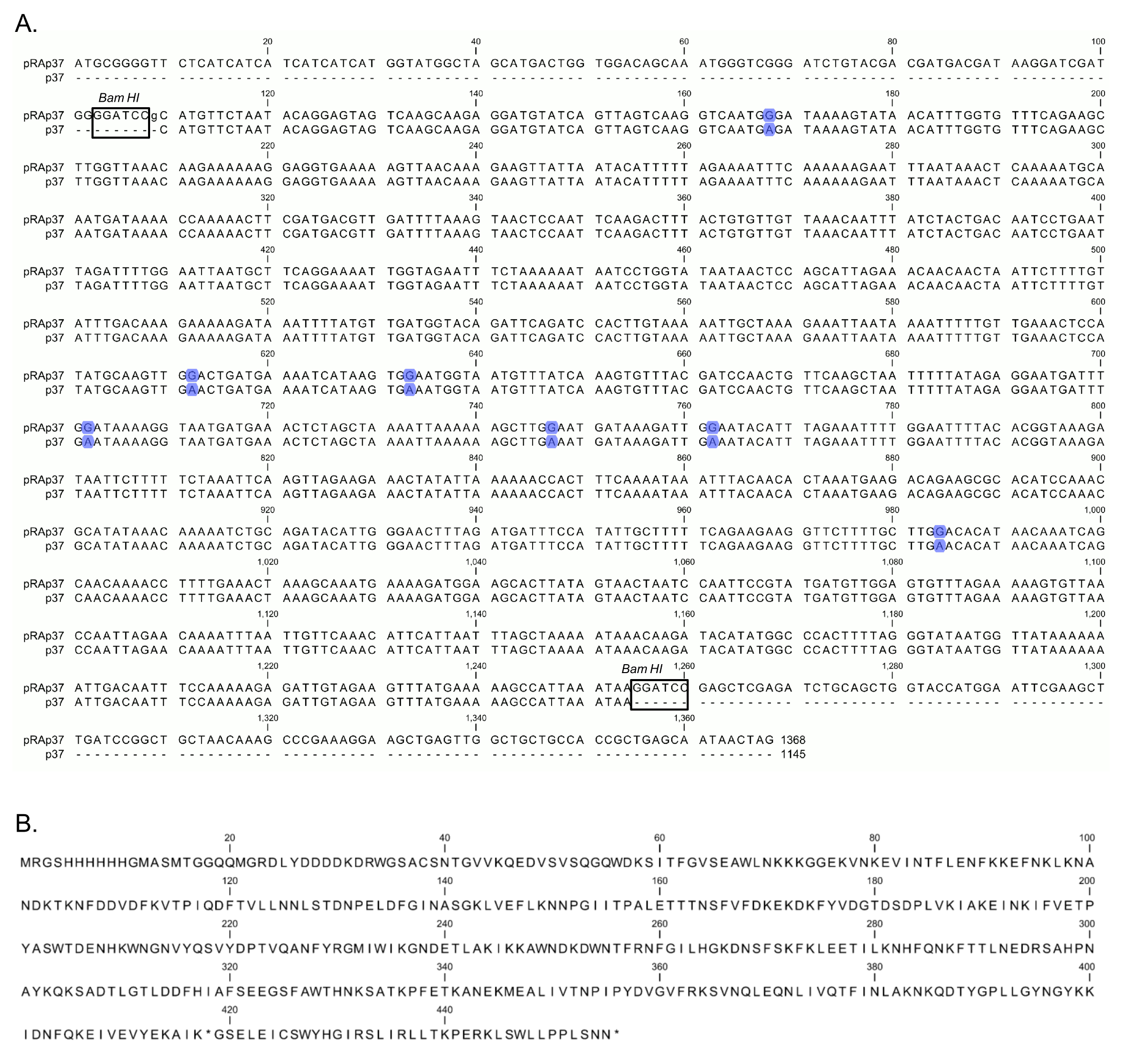

Supplement: S1 Fig — The p37 gene, excluding the signal sequence, was cloned into the BamHI cut site (green) of pRSET A (A). TGA codons mutated to TGG for tryptophan (W) expression in E. coli are indicated in blue. An extra base pair ‘g’ (yellow) was inserted to ensure p37 was in frame for correct protein synthesis (B). Basic sequence alignment and analysis was performed utilising the program CLC Sequence Viewer 6 (Version 6.8.1). (TIF) [file pone.0140753.s001.tif]

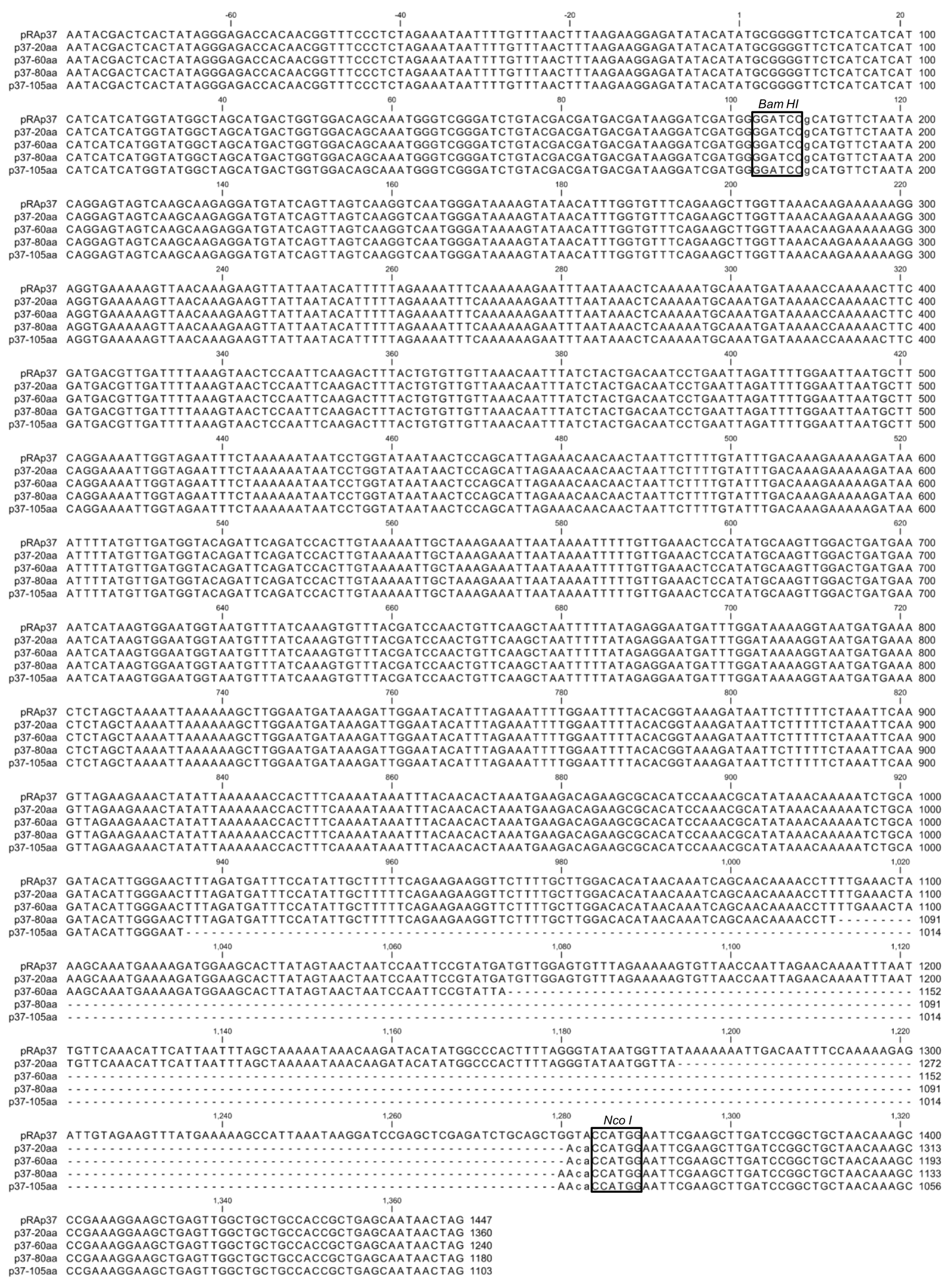

Supplement: S2 Fig — The locations of the forward and reverse primers are highlighted in blue. Basic sequence alignments and analysis was performed utilising the program CLC Sequence Viewer 6 (Version 6.8.1). (TIF) [file pone.0140753.s002.tif]

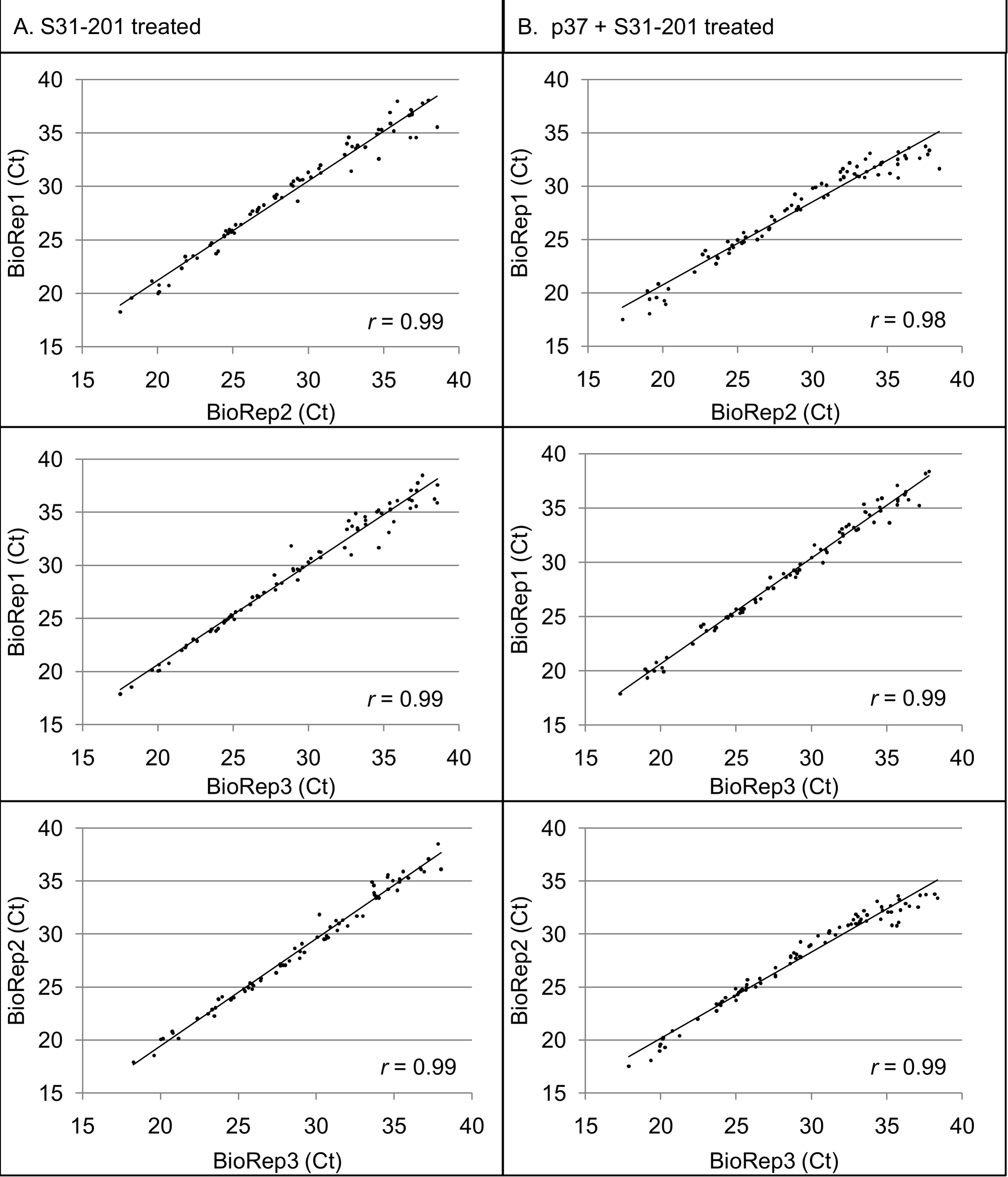

Supplement: S4 Fig — Correlation plots of 96 gene Ct values between the triplicate Profiler array biological replicates (BioRep1, 2 and 3) for S31-201 treated NIH3T3 cells (A) and 25 μg ml-1 p37 treated NIH3T3 cells, pre-treated with S31-201 (B) (N = 96). Strong Pearson correlation coefficients (r), determined by the linear regression, are indicated. (TIF) [file pone.0140753.s004.tif]

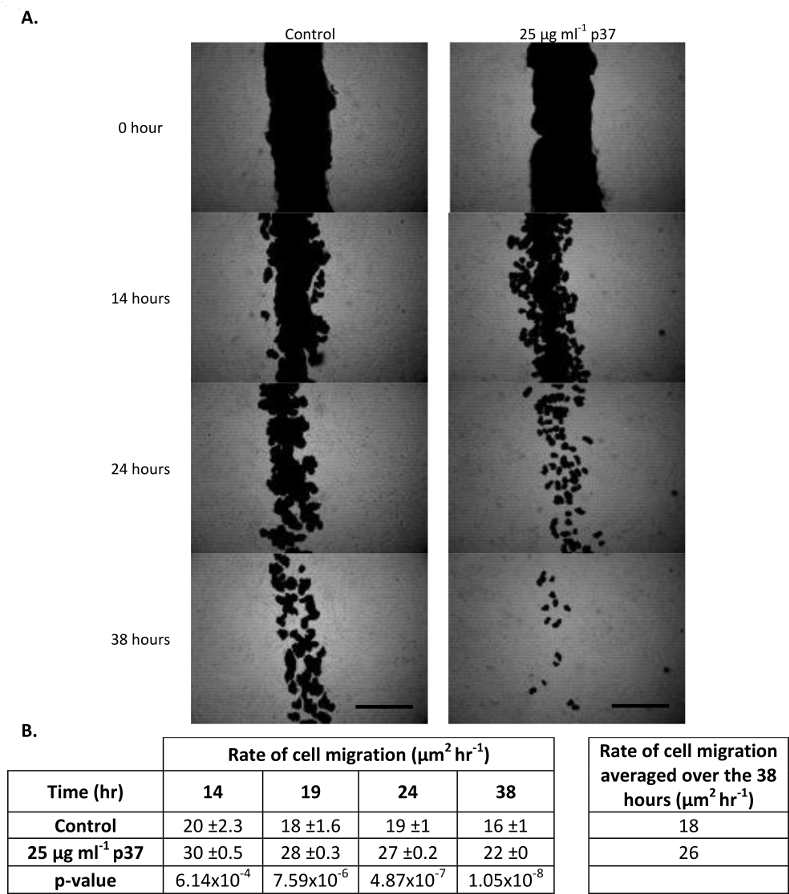

Supplement: S5 Fig — (TIF) [file pone.0140753.s005.tif]

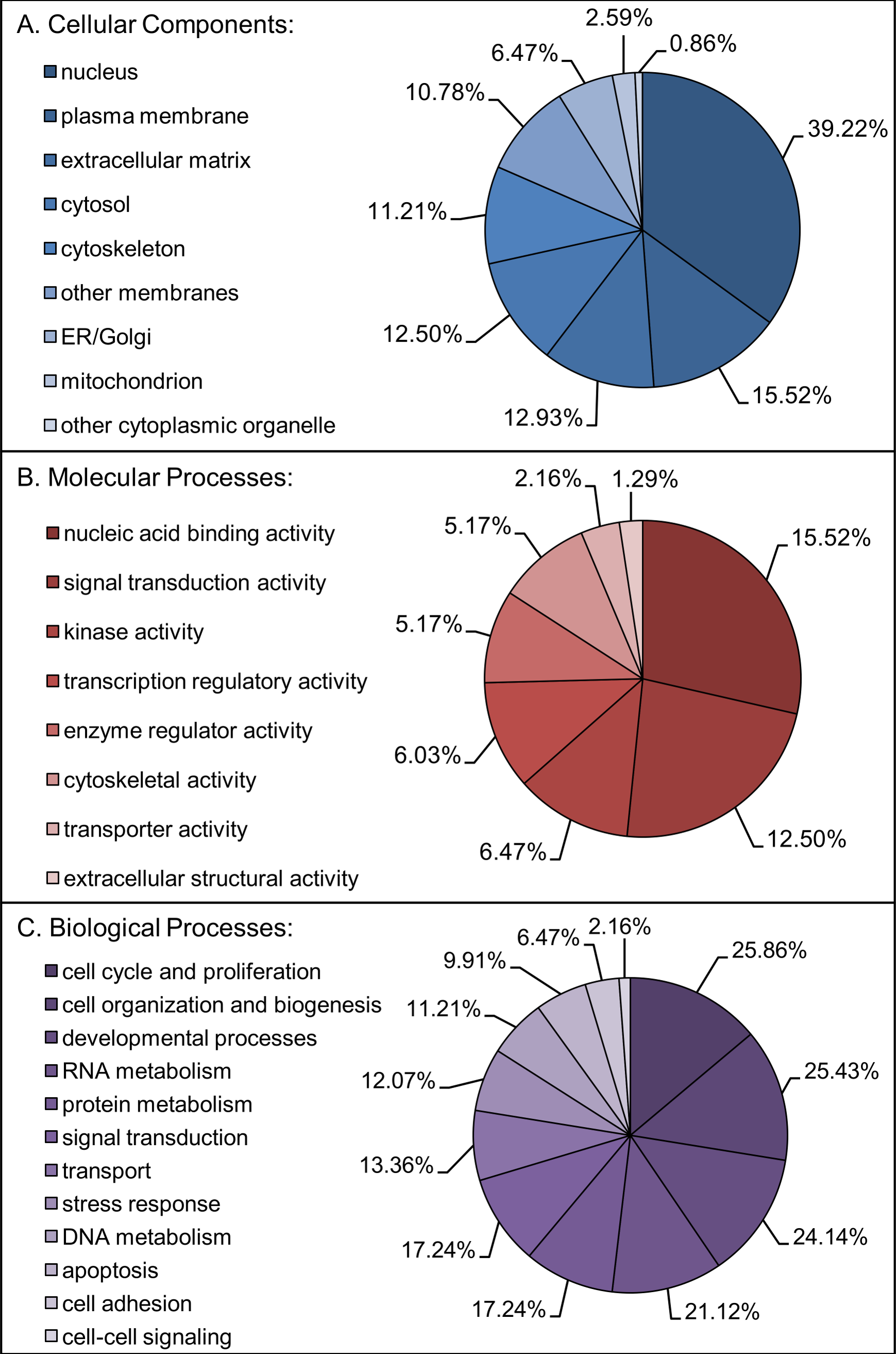

Supplement: S6 Fig — Cellular components (a), molecular processes (b) and biological processes (c) regulated by the genes significantly upregulated by p37, assessed by Gene Ontology (GO). GO classifications are extracted from the Mouse Genome Informatics Database. Note: An individual protein can be associated with more than one GOterm. (TIF) [file pone.0140753.s006.tif]

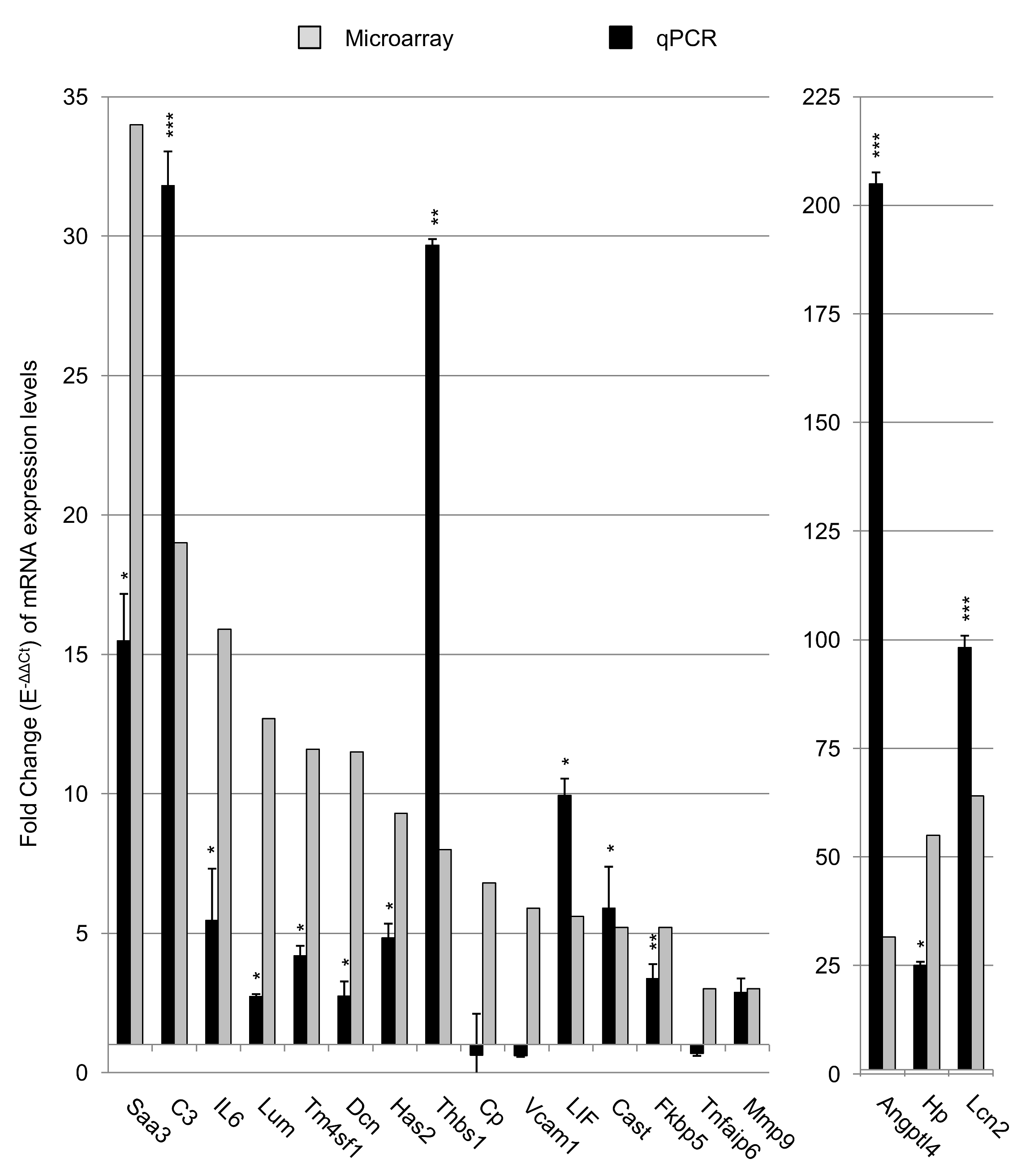

Supplement: S7 Fig — NIH3T3 fibroblasts were treated with 15 μg ml-1 of purified p37 for 24 hours. Quantitative PCR (qPCR) was used to validate p37-induced expression of 18 genes (p ≤ 0.001, fold change ≥ 3) identified in the microarray analysis using Affymetrix Mouse Genome 430 2.0 Arrays. Black bars represent Microarray mRNA levels expressed as absolute fold change (treated vs. untreated); p-values ≤ 0.001. Gray bars represent qPCR mRNA levels expressed as fold change (E-ΔΔCt) relative to untreated controls and normalized to the reference genes GAPDH and βactin. Significant differences between treated and untreated cells were calculated by ANOVA analysis (*p<0.05, **p<0.01, ***p<0.001). (TIF) [file pone.0140753.s007.tif]

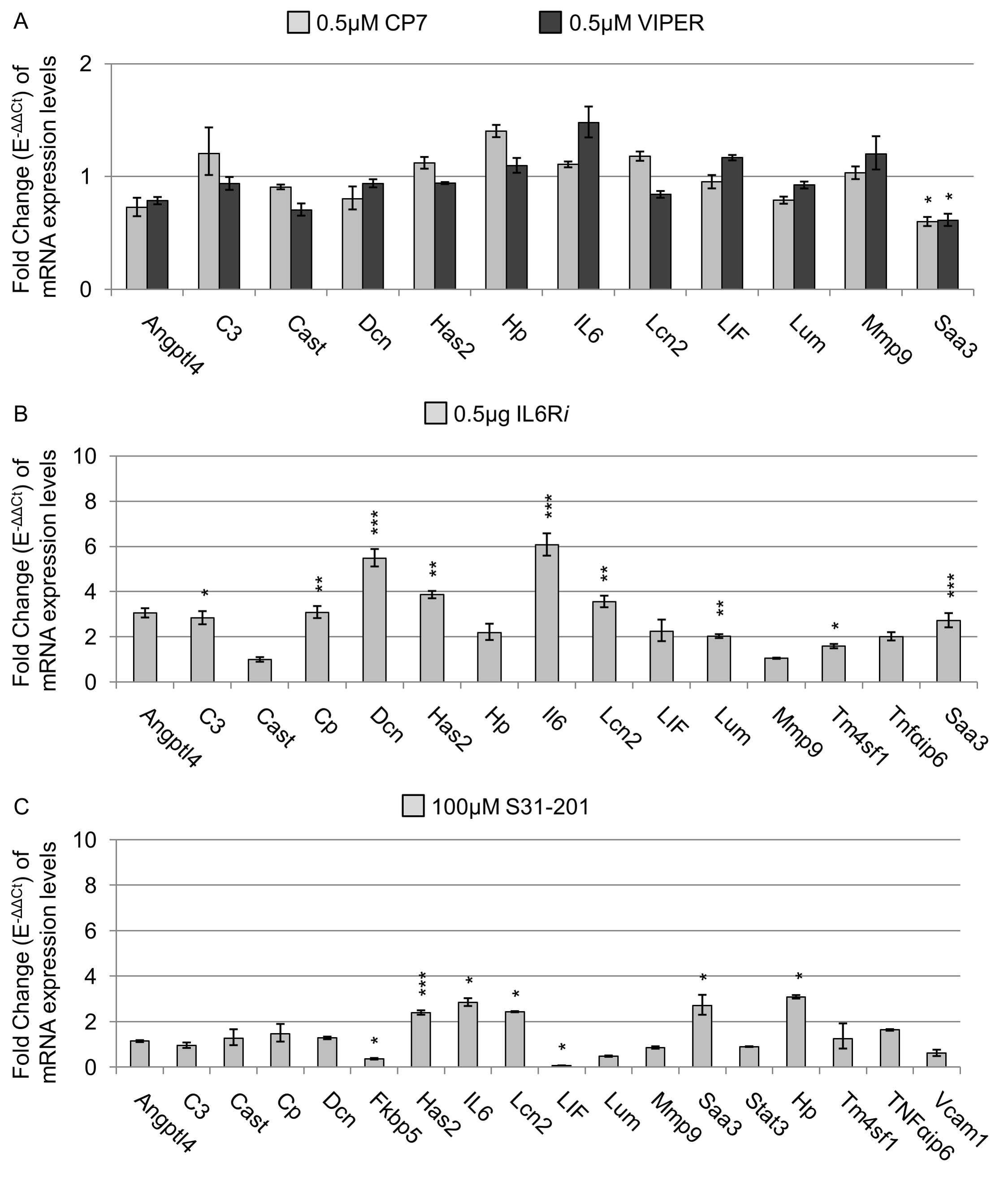

Supplement: S8 Fig — qPCR analysis of NIH3T3 fibroblasts treated with 0.5 μM VIPER or CP7 for 26 hours (A), 0.1 μg ml-1 IL6R antibody inhibitor (IL6Ri) for 25 hours (B) or 100 μM S31-201 for 48 hours (C). Significant differences between treated and untreated cells were calculated by ANOVA analysis (*p<0.05, **p<0.01, ***p<0.001). (TIF) [file pone.0140753.s008.tif]

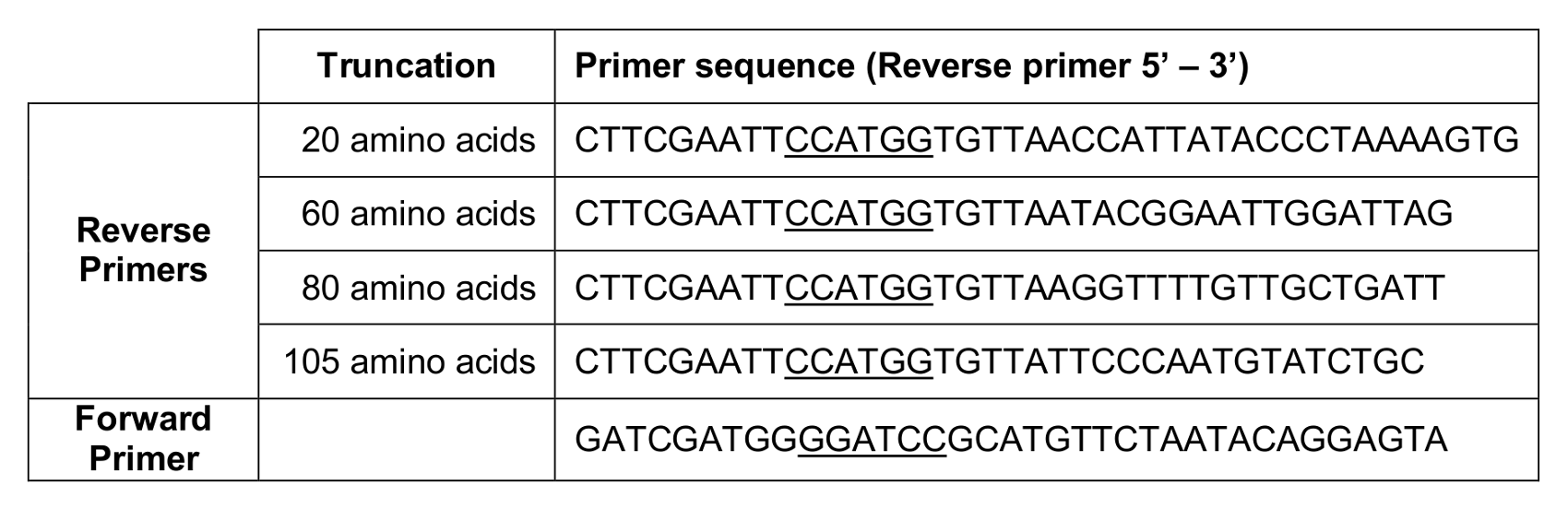

Supplement: S1 Table — NcoI restriction enzyme cut site in the reverse primers and BamHI in the forward primers are indicated by underline. (TIF) [file pone.0140753.s009.tif]

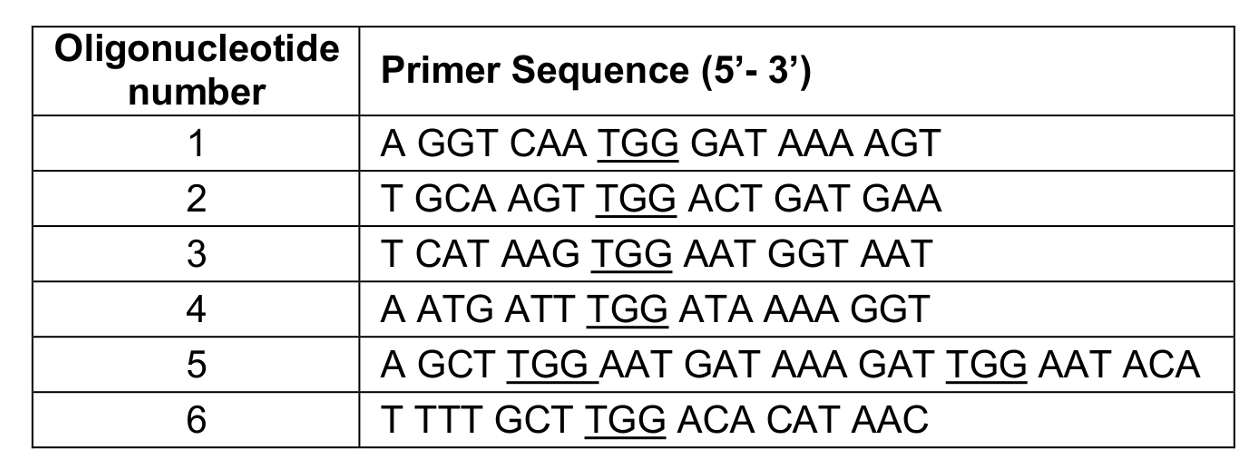

Supplement: S2 Table — (TIF) [file pone.0140753.s010.tif]

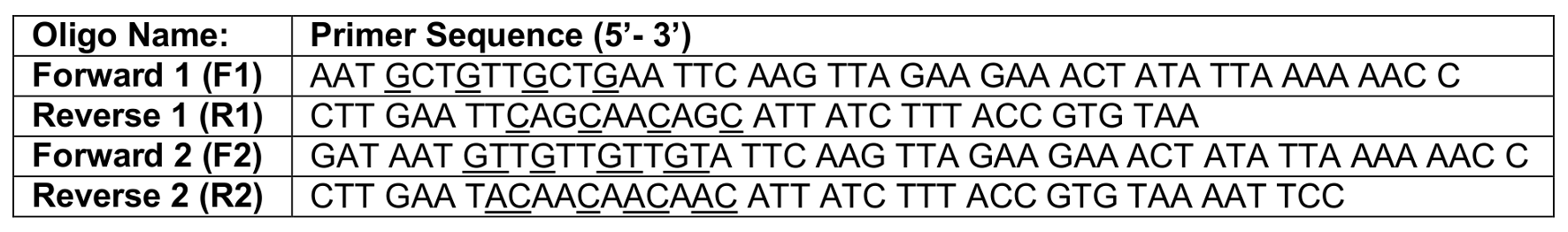

Supplement: S3 Table — The bold base pairs are those introducing point mutations to the p37 gene. Oligonucleotides Forward 1 and Reverse 1 were used in the first PCR and Forward 2 and Reverse 2 were used in the second PCR. (TIF) [file pone.0140753.s011.tif]

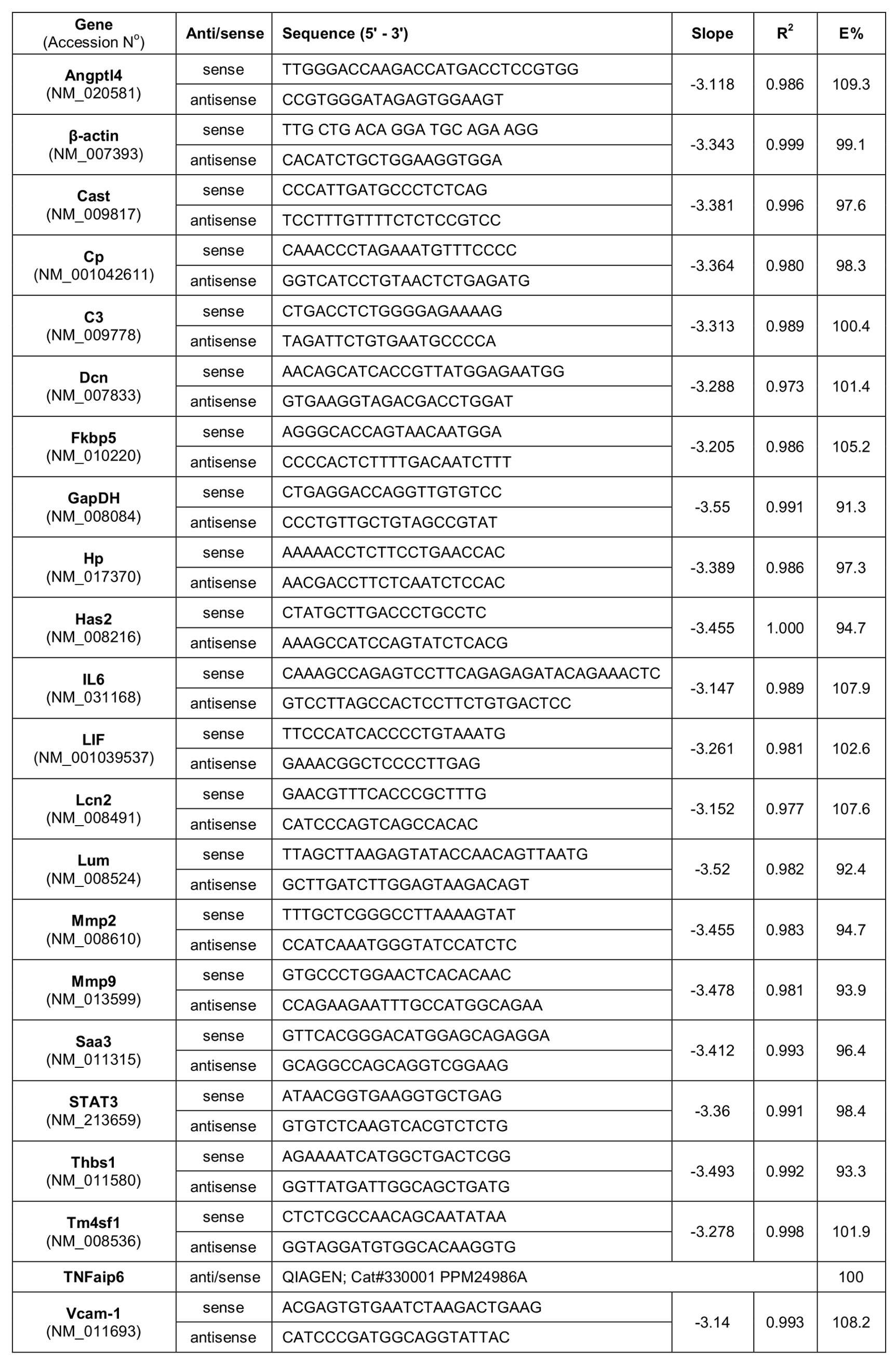

Supplement: S4 Table — (TIF) [file pone.0140753.s012.tif]

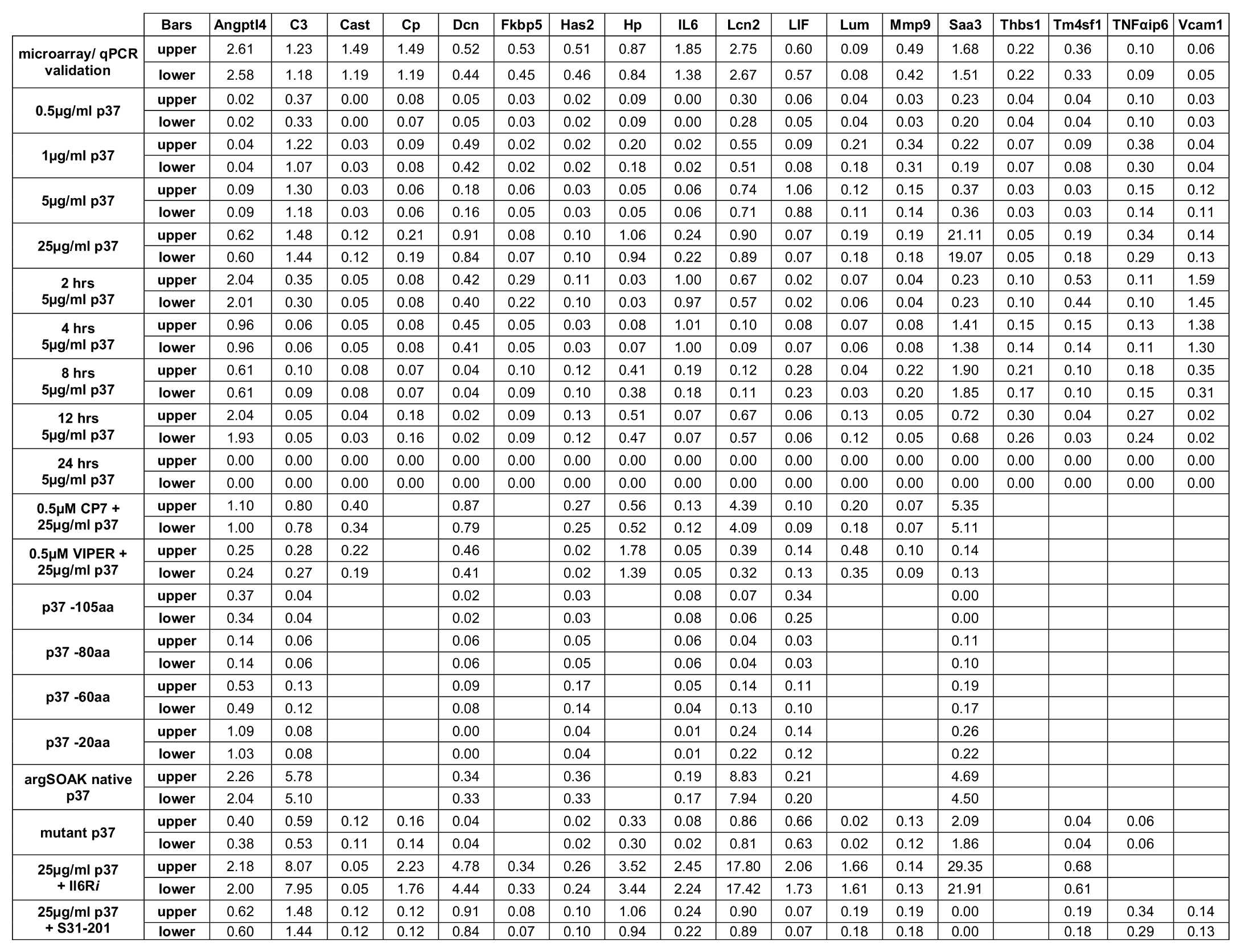

Supplement: S5 Table — (TIF) [file pone.0140753.s013.tif]

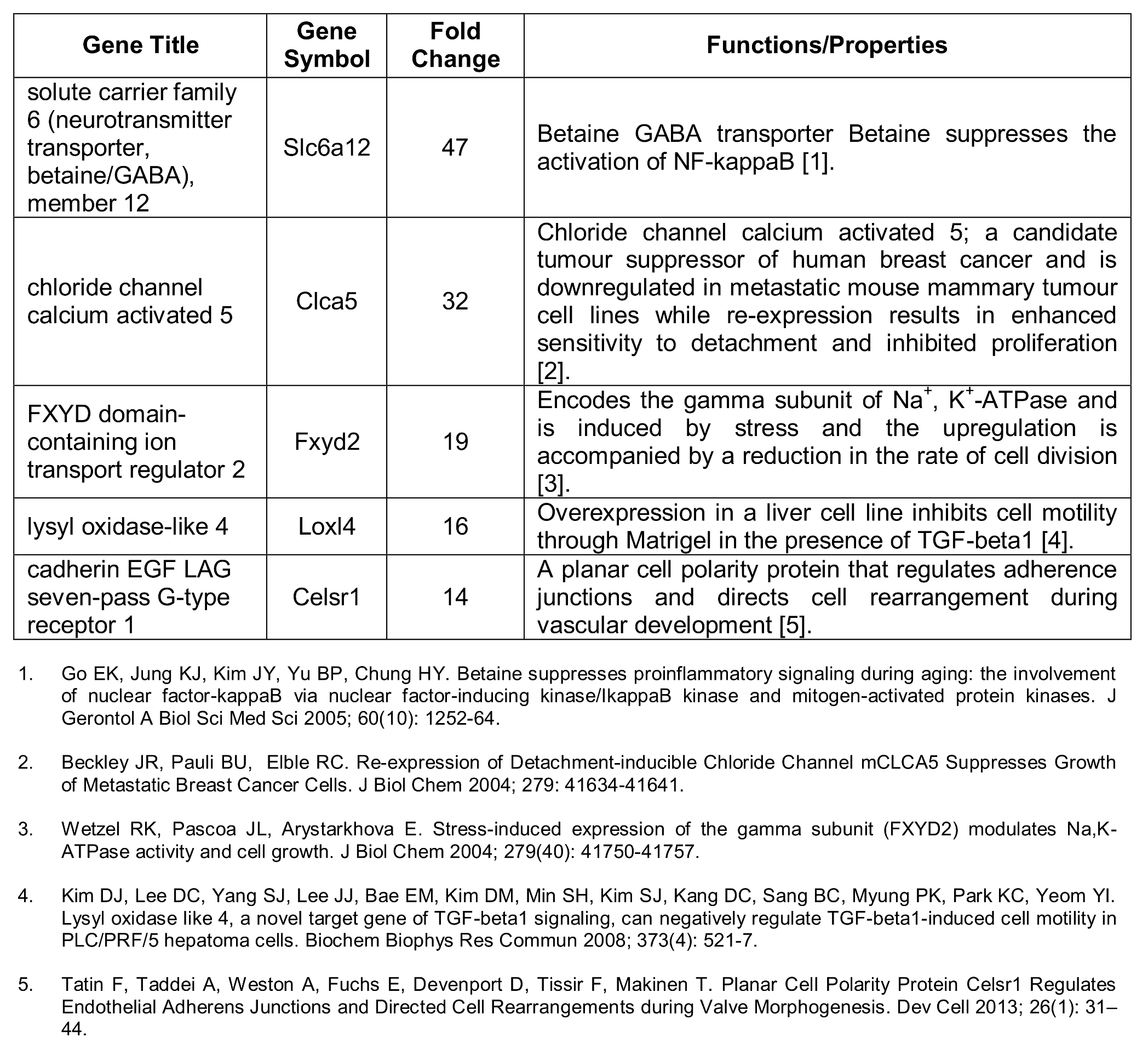

Supplement: S8 Table — (TIF) [file pone.0140753.s016.tif]
