# Supplementary material for: The Mycoplasma hyorhinis p37 Protein Rapidly Induces Genes in Fibroblasts Associated with Inflammation and Cancer
Source: PLoS One. 2015 Oct 29;10(10):e0140753. doi: 10.1371/journal.pone.0140753 (PMC4626034; doi:10.1371/journal.pone.0140753)
Supplement: S6 Table — The dataset consists of 288 genes significantly upregulated by ≥ 3 fold with a p-value of ≤ 0.001. Genes chosen for further study are indicated in bold. (PDF) [file pone.0140753.s014.pdf]

Affymetrix Probe Set ID	Gene Symbol	Gene Title	p-value	Fold Change (Up)
**1427747_a_at**	**Lcn2**	**lipocalin 2**	**1.9E-04**	**64**
**1448881_at**	**Hp**	**haptoglobin**	**1.0E-04**	**55**
**1450826_a_at**	**Saa3**	**serum amyloid A 3**	**8.9E-05**	**34**
**1417130_s_at**	**Angptl4**	**angiopoietin-like 4**	**2.4E-04**	**31**
**1423954_at**	**C3**	**complement component 3**	**8.6E-05**	**19**
**1450297_at**	**Il6**	**interleukin 6**	**5.5E-05**	**16**
**1423607_at**	**Lum**	**lumican**	**4.9E-05**	**13**
**1439925_at**	**Tm4sf1**	**transmembrane 4 superfamily member 1**	**1.1E-04**	**12**
**1449368_at**	**Dcn**	**decorin**	**3.1E-05**	**11**
**1449169_at**	**Has2**	**hyaluronan synthase 2**	**4.1E-04**	**9**
1450854_at	Pa2g4	proliferation-associated 2G4	1.3E-04	9
1460238_at	Msln	mesothelin	3.6E-04	9
1420142_s_at	Pa2g4	proliferation-associated 2G4	4.3E-04	9
1438009_at	RP23-480B19.10	similar to histone 2a	7.0E-04	9
1418678_at	Has2	hyaluronan synthase 2	3.5E-05	8
1449017_at	Nutf2	nuclear transport factor 2	1.3E-04	8
1460302_at	Thbs1	thrombospondin 1 /// similar to thrombospondin 1	5.1E-04	8
1416721_s_at	Sfrs6	splicing factor, arginine/serine-rich 6	3.7E-04	8
1431375_s_at	Parva	parvin, alpha	1.7E-04	8
1423282_at	Pitpna	phosphatidylinositol transfer protein, alpha	1.0E-04	7
1455494_at	Col1a1	procollagen, type I, alpha 1	1.8E-04	7
**1421811_at**	**Thbs1**	**thrombospondin 1**	**5.1E-04**	**7**
**1448735_at**	**Cp**	**ceruloplasmin**	**3.0E-04**	**7**
1455505_at	Gatad2a	GATA zinc finger domain containing 2A	1.2E-06	7
1431213_a_at	LOC100041156	hypothetical protein LOC100041156 /// hypothetical protein LOC100041932	7.6E-04	7
1422535_at	Ccne2	cyclin E2	6.6E-04	7
1449550_at	Myo1c	myosin IC	1.2E-05	6
1438658_a_at	Edg3	endothelial differentiation, sphingolipid G-protein-coupled receptor, 3	9.3E-05	6
1439516_at	2610201A13Rik	RIKEN cDNA 2610201A13 gene	6.4E-04	6
1437611_x_at	Kif2c	kinesin family member 2C	1.9E-04	6
1416720_at	Sfrs6	splicing factor, arginine/serine-rich 6	1.5E-04	6
1430139_at	Hells	helicase, lymphoid specific	1.7E-04	6
1435338_at	Cdk6	cyclin-dependent kinase 6	5.1E-04	6
1416155_at	Hmgb3	high mobility group box 3	1.2E-04	6
1417586_at	Timeless	timeless homolog (Drosophila)	5.7E-05	6
1447898_s_at	Sfrs6	splicing factor, arginine/serine-rich 6	2.4E-04	6
1420380_at	Ccl2	chemokine (C-C motif) ligand 2	7.3E-06	6
1417625_s_at	Cxcr7	chemokine (C-X-C motif) receptor 7	3.1E-05	6
**1448162_at**	**Vcam1**	**vascular cell adhesion molecule 1**	**1.2E-04**	**6**
1423422_at	Asb4	ankyrin repeat and SOCS box-containing protein 4	1.8E-04	6
1434089_at	Synpo	synaptopodin	7.2E-04	6
1435047_at		Transcribed locus	2.0E-05	6
1435315_s_at	2900034E22Rik	RIKEN cDNA 2900034E22 gene	1.2E-04	6
**1421207_at**	**LIF**	**leukemia inhibitory factor**	**4.1E-04**	**6**
1448961_at	Plscr2	phospholipid scramblase 2	8.4E-05	6
1455529_at	Mex3a	mex3 homolog A (C. elegans)	2.0E-04	6
1452912_at	2600005O03Rik	RIKEN cDNA 2600005O03 gene	1.4E-04	6
1460212_at	Gnat1	guanine nucleotide binding protein, alpha transducing 1	6.3E-05	5

1455287_at	Cdk6	cyclin-dependent kinase 6	1.4E-04	5
1426529_a_at	Tagln2	transgelin 2	4.7E-05	5
1419749_at	Trdmt1	tRNA aspartic acid methyltransferase 1	2.1E-04	5
1435000_at	Gspt1	G1 to S phase transition 1	1.0E-04	5
1417495_x_at	Cp	ceruloplasmin	6.8E-04	5
1417155_at	Mycn	v-myc myelocytomatosis viral related oncogene, neuroblastoma derived (avian)	6.5E-04	5
1417494_a_at	Cp	ceruloplasmin	7.6E-04	5
1428069_at	Cdca7	cell division cycle associated 7	9.4E-04	5
**1416125_at**	**Fkbp5**	**FK506 binding protein 5**	**5.1E-07**	**5**
1419182_at	Svep1	sushi, von Willebrand factor type A, EGF and pentraxin domain containing 1	2.0E-04	5
**1435972_at**	**Cast**	**calpastatin**	**6.9E-05**	**5**
1449893_a_at	Lrig1	leucine-rich repeats and immunoglobulin-like domains 1	3.2E-04	5
1420980_at	Pak1	p21 (CDKN1A)-activated kinase 1	3.6E-05	5
1439407_x_at	Tagln2	transgelin 2	1.1E-04	5
1439191_at		0 day neonate eyeball cDNA, RIKEN full-length enriched library, clone:E130107G13 product:histocompatibility 2, T region locus 18, full insert sequence	4.9E-04	5
1447369_at	1190005F20Rik	RIKEN cDNA 1190005F20 gene	5.0E-05	5
1434552_at	Wdr77	WD repeat domain 77	5.1E-05	5
1433919_at	Asb4	ankyrin repeat and SOCS box-containing protein 4	1.4E-04	5
1448377_at	Slpi	secretory leukocyte peptidase inhibitor	4.3E-04	5
1437173_at	Edg3	endothelial differentiation, sphingolipid G-protein-coupled receptor, 3	2.0E-04	5
1419869_s_at	Hdlbp	high density lipoprotein (HDL) binding protein	6.7E-05	5
1437071_at	Eif1ay	eukaryotic translation initiation factor 1A, Y-linked	2.0E-04	5
1453181_x_at	Plscr1	phospholipid scramblase 1	7.2E-04	5
1424949_at	Huwe1	HECT, UBA and WWE domain containing 1	1.6E-04	5
1450070_s_at	Pak1	p21 (CDKN1A)-activated kinase 1	8.7E-05	5
1444531_at		Adult male urinary bladder cDNA, RIKEN full-length enriched library, clone:9530055M18 product:unclassifiable, full insert sequence	2.2E-04	5
1415810_at	Uhrf1	ubiquitin-like, containing PHD and RING finger domains, 1	4.9E-04	5
1428819_at	Mapre1	microtubule-associated protein, RP/EB family, member 1	1.5E-05	5
1453708_a_at	Gsto2	glutathione S-transferase omega 2	2.8E-04	5
1439648_at	Anln	anillin, actin binding protein (scraps homolog, Drosophila)	9.8E-04	5
1448326_a_at	Crabp1	cellular retinoic acid binding protein I	5.9E-04	5
1434555_at	Anp32a	acidic (leucine-rich) nuclear phosphoprotein 32 family, member A	6.4E-05	5
1421228_at	Ccl7	chemokine (C-C motif) ligand 7	1.4E-04	5
1416129_at	Errfi1	ERBB receptor feedback inhibitor 1	4.8E-04	5
1427883_a_at	Col3a1	procollagen, type III, alpha 1	4.9E-04	5
1441855_x_at	Cxcl1	chemokine (C-X-C motif) ligand 1	1.5E-04	5
1416227_at	Arpc1b /// EG434782	actin related protein 2/3 complex, subunit 1B /// predicted gene, EG434782	1.6E-04	5
1450920_at	Ccnb2	cyclin B2	4.2E-04	5
1417587_at	Timeless	timeless homolog (Drosophila)	3.2E-04	5
1448734_at	Cp	ceruloplasmin	1.4E-04	5
1436349_at	2700094K13Rik	RIKEN cDNA 2700094K13 gene	8.9E-04	5
1429527_a_at	Plscr1	phospholipid scramblase 1	4.7E-04	4
1416258_at	Tk1	thymidine kinase 1	7.5E-05	4
1418255_s_at	Srf	serum response factor	1.9E-05	4
1416030_a_at	Mcm7	minichromosome maintenance deficient 7 (S. cerevisiae)	1.1E-04	4
1417019_a_at	Cdc6	cell division cycle 6 homolog (S. cerevisiae)	2.2E-04	4
1434311_at	Cnot6l	CCR4-NOT transcription complex, subunit 6-like	4.7E-05	4
1435977_at	Hdgfrp3	hepatoma-derived growth factor, related protein 3	2.7E-04	4

1438571_at	Bub1	Budding uninhibited by benzimidazoles 1 homolog (S. cerevisiae)	1.9E-04	4
1436036_at	Whsc1	Wolf-Hirschhorn syndrome candidate 1 (human)	2.0E-05	4
1455591_at			3.6E-05	4
1446791_at		Transcribed locus	3.3E-04	4
1451413_at	Cast	calpastatin	1.2E-04	4
1426897_at	Rcc2	regulator of chromosome condensation 2	4.9E-04	4
1455060_at	G3bp1	Ras-GTPase-activating protein SH3-domain binding protein 1	2.4E-05	4
1454946_at	Mybl2	myeloblastosis oncogene-like 2	5.2E-04	4
1440884_s_at	A530047J11Rik	RIKEN cDNA A530047J11 gene	6.7E-04	4
1449060_at	Kif2c	kinesin family member 2C	7.8E-06	4
1434210_s_at	Lrig1	leucine-rich repeats and immunoglobulin-like domains 1	3.0E-04	4
1455715_at	LOC100038746	hypothetical LOC100038746	1.7E-04	4
1416715_at	Gjb3	gap junction membrane channel protein beta 3	1.9E-04	4
1434850_at	Iqgap3	IQ motif containing GTPase activating protein 3	5.8E-04	4
1416593_at	Glrx	glutaredoxin	9.8E-05	4
1415996_at	Txnip	thioredoxin interacting protein	3.8E-04	4
1436186_at	E2f8	E2F transcription factor 8	8.6E-04	4
1428412_at	Tm9sf3	transmembrane 9 superfamily member 3	7.4E-04	4
1419816_s_at	Errfi1	ERBB receptor feedback inhibitor 1	4.8E-04	4
1428280_at	Fip1l1	FIP1 like 1 (S. cerevisiae)	1.9E-06	4
1415989_at	Vcam1	vascular cell adhesion molecule 1	2.7E-05	4
1416120_at	Rrm2	ribonucleotide reductase M2	1.1E-04	4
1452872_at	Ank3	ankyrin 3, epithelial	3.1E-04	4
1457687_at	Bcl2	B-cell leukemia/lymphoma 2	9.1E-04	4
1448584_at	Rsrc1	arginine/serine-rich coiled-coil 1	1.1E-05	4
1454995_at	Ddah1	dimethylarginine dimethylaminohydrolase 1	2.4E-05	4
1448226_at	Rrm2	ribonucleotide reductase M2	8.7E-04	4
1460208_at	Fbn1	fibrillin 1	7.5E-05	4
1452348_s_at	Ifi203	interferon activated gene 203	3.9E-04	4
1418402_at	Adam19	a disintegrin and metallopeptidase domain 19 (meltrin beta)	7.7E-05	4
1416031_s_at	Mcm7	minichromosome maintenance deficient 7 (S. cerevisiae)	2.0E-04	4
1422692_at	Sub1	SUB1 homolog (S. cerevisiae)	4.6E-04	4
1418176_at	Vdr	vitamin D receptor	5.9E-04	4
1428142_at	Etv5	ets variant gene 5	4.2E-04	4
1438404_at	Rnf144	ring finger protein 144	6.5E-05	4
1436555_at	Slc7a2	solute carrier family 7 (cationic amino acid transporter, y+ system), member 2	2.4E-04	4
1419153_at	2810417H13Rik	RIKEN cDNA 2810417H13 gene	5.9E-05	4
1425528_at	Prrx1	paired related homeobox 1	5.5E-04	4
1417821_at	D17H6S56E-5	DNA segment, Chr 17, human D6S56E 5	3.0E-05	4
1434437_x_at	Rrm2	ribonucleotide reductase M2	3.9E-04	4
1416073_a_at	Nup85	nucleoporin 85	6.5E-05	4
1448669_at	Dkk3	dickkopf homolog 3 (Xenopus laevis)	5.0E-04	4
1435384_at	Ube2n	ubiquitin-conjugating enzyme E2N	9.0E-04	4
1417910_at	Ccna2	cyclin A2	1.5E-04	4
1440396_at			2.0E-04	4
1429095_at	Cenpp	centromere protein P	3.1E-04	4
1419639_at	Efnb2	ephrin B2	9.8E-05	4
1418175_at	Vdr	vitamin D receptor	8.4E-05	4
1434570_at	AK122525	cDNA sequence AK122525	4.4E-04	4
1452098_at	Chtf18	CTF18, chromosome transmission fidelity factor 18 homolog (S. cerevisiae)	8.4E-05	4

1429076_a_at	Gdpd2	glycerophosphodiester phosphodiesterase domain containing 2	8.5E-04	4
1449708_s_at	Chek1	checkpoint kinase 1 homolog (S. pombe)	1.5E-04	4
1448627_s_at	Pbk	PDZ binding kinase	2.9E-04	4
1417971_at	Nrm	nurim (nuclear envelope membrane protein)	4.9E-04	4
1419015_at	Wisp2	WNT1 inducible signalling pathway protein 2	5.5E-04	4
1450677_at	Chek1	checkpoint kinase 1 homolog (S. pombe)	5.4E-04	4
1455899_x_at	Socs3	suppressor of cytokine signalling 3	2.7E-04	4
1439901_at		Transcribed locus	5.4E-04	4
1455160_at	2610203C20Rik	RIKEN cDNA 2610203C20 gene	8.9E-05	4
1416454_s_at	Acta2	actin, alpha 2, smooth muscle, aorta	7.2E-04	4
1457644_s_at	Cxcl1	chemokine (C-X-C motif) ligand 1	9.6E-05	4
1455680_at	9630025H16Rik	RIKEN cDNA 9630025H16 gene	9.8E-04	4
1439899_at	Galnt13	UDP-N-acetyl-alpha-D-galactosamine:polypeptide N-acetylgalactosaminyltransferase 13	3.2E-04	4
1460415_a_at	Cd40	CD40 antigen	8.6E-04	4
1416953_at	Ctgf	connective tissue growth factor	4.3E-04	4
1416017_at	Copg	coatomer protein complex, subunit gamma	4.6E-04	4
1427348_at	Zc3h12a	zinc finger CCCH type containing 12A	7.8E-04	4
1431087_at	Spc24	SPC24, NDC80 kinetochore complex component, homolog (S. cerevisiae)	2.8E-05	4
1436917_s_at	Gpsm1	G-protein signalling modulator 1 (AGS3-like, C. elegans)	1.4E-04	4
1436723_at	Cenpi	centromere protein I	4.3E-04	4
1433507_a_at	Hmgn2	high mobility group nucleosomal binding domain 2	8.2E-04	4
1416042_s_at	Nasp	nuclear autoantigenic sperm protein (histone-binding)	2.2E-04	4
1456221_at	ENSMUSG00000074134	Predicted gene, ENSMUSG00000074134	2.1E-04	4
1452881_at	Gins2	GINS complex subunit 2 (Psf2 homolog)	1.0E-04	4
1423877_at	Chaf1b	chromatin assembly factor 1, subunit B (p60)	3.7E-04	4
1419209_at	Cxcl1	chemokine (C-X-C motif) ligand 1	1.3E-04	4
1419603_at	Ifi204	interferon activated gene 204	1.4E-04	4
1449314_at	Zfpm2	zinc finger protein, multitype 2	3.4E-04	4
1416592_at	Glrx	glutaredoxin	2.8E-04	4
1452534_a_at	Hmgb2	high mobility group box 2	8.1E-04	4
1423775_s_at	Prc1	protein regulator of cytokinesis 1	5.0E-04	4
1426631_at	Pus7	pseudouridylate synthase 7 homolog (S. cerevisiae)	1.5E-04	4
1439740_s_at	Uck2	uridine-cytidine kinase 2	5.3E-04	3
1450496_a_at	2810433K01Rik	RIKEN cDNA 2810433K01 gene	2.2E-04	3
1453748_a_at	Kif23	kinesin family member 23	4.1E-05	3
1426864_a_at	Ncam1	neural cell adhesion molecule 1	4.3E-05	3
1455173_at	Gspt1	G1 to S phase transition 1	8.0E-05	3
1435170_at	Tsr2	TSR2, 20S rRNA accumulation, homolog (S. cerevisiae)	3.7E-04	3
1424991_s_at	Tyms /// Tyms-ps	thymidylate synthase /// thymidylate synthase, pseudogene	2.7E-04	3
1424128_x_at	Aurkb	aurora kinase B	3.9E-05	3
1455352_at	AU023006	expressed sequence AU023006	2.5E-04	3
1424759_at	Arrdc4	arrestin domain containing 4	2.6E-04	3
1415945_at	Mcm5	minichromosome maintenance deficient 5, cell division cycle 46 (S. cerevisiae)	4.9E-04	3
1457614_at		Transcribed locus	7.5E-04	3
1435797_at	D5Wsu178e	DNA segment, Chr 5, Wayne State University 178, expressed	6.4E-04	3
1439394_x_at	Cdc20	cell division cycle 20 homolog (S. cerevisiae)	4.2E-04	3
1440169_x_at	Ifnar2	interferon (alpha and beta) receptor 2	5.6E-05	3
1424046_at	Bub1	budding uninhibited by benzimidazoles 1 homolog (S. cerevisiae)	2.7E-04	3
1452387_a_at	Amotl2	angiomotin like 2	4.2E-04	3

1451246_s_at	Aurkb	aurora kinase B	6.9E-06	3
1435306_a_at	Kif11	kinesin family member 11	2.2E-04	3
1436211_at	Thoc4	THO complex 4	3.3E-04	3
1428187_at	Cd47	CD47 antigen (Rh-related antigen, integrin-associated signal transducer)	4.4E-05	3
1439741_x_at	Uck2	uridine-cytidine kinase 2	6.9E-04	3
1435743_at	Klhl23	kelch-like 23 (Drosophila)	2.4E-04	3
1435594_at	Arl6ip2	ADP-ribosylation factor-like 6 interacting protein 2	9.9E-04	3
1433234_at	4930424E08Rik	RIKEN cDNA 4930424E08 gene	9.7E-04	3
1422628_at	4632417K18Rik	RIKEN cDNA 4632417K18 gene	9.0E-05	3
1436585_at	BB182297	expressed sequence BB182297	9.2E-05	3
1451206_s_at	Pscdbp	pleckstrin homology, Sec7 and coiled-coil domains, binding protein	4.1E-04	3
1436217_at	Zfp148	zinc finger protein 148	4.9E-04	3
1426818_at	Arrdc4	arrestin domain containing 4	1.3E-04	3
1456280_at	Clspn	claspin homolog (Xenopus laevis)	2.1E-04	3
1423714_at	Asf1b	ASF1 anti-silencing function 1 homolog B (S. cerevisiae)	1.2E-04	3
1432179_x_at	2810433K01Rik	RIKEN cDNA 2810433K01 gene	5.9E-04	3
1454788_at	Arl4c /// LOC632433	ADP-ribosylation factor-like 4C /// similar to ADP-ribosylation factor-like protein 7	3.1E-04	3
1426909_at	Uck2	uridine-cytidine kinase 2	4.0E-04	3
1460009_at		Transcribed locus	5.8E-04	3
1417938_at	Rad51ap1	RAD51 associated protein 1	3.0E-04	3
1423596_at	Nek6	NIMA (never in mitosis gene a)-related expressed kinase 6	2.1E-04	3
1428713_s_at	Gins2	GINS complex subunit 2 (Psf2 homolog)	6.1E-04	3
1426652_at	Mcm3	minichromosome maintenance deficient 3 (S. cerevisiae)	8.1E-05	3
1430530_s_at	Nmral1	NmrA-like family domain containing 1	1.1E-04	3
1439263_at	LOC14210	hypothetical LOC14210	7.7E-05	3
1419554_at	Cd47	CD47 antigen (Rh-related antigen, integrin-associated signal transducer)	2.5E-05	3
1451077_at	Rpl5	ribosomal protein L5	4.3E-04	3
1452314_at	Kif11	kinesin family member 11	1.6E-05	3
1431751_a_at	Mpped2	metallophosphoesterase domain containing 2	4.0E-05	3
1455737_at	C030002B11Rik	RIKEN cDNA C030002B11 gene	6.7E-04	3
1419838_s_at	Plk4	polo-like kinase 4 (Drosophila)	1.6E-04	3
1434232_a_at	2610030H06Rik	RIKEN cDNA 2610030H06 gene	9.3E-05	3
1424524_at	1200002N14Rik	RIKEN cDNA 1200002N14 gene	6.7E-04	3
1416664_at	Cdc20	cell division cycle 20 homolog (S. cerevisiae)	3.9E-04	3
1423805_at	Dab2	disabled homolog 2 (Drosophila)	5.6E-05	3
1448777_at	Mcm2	minichromosome maintenance deficient 2 mitotin (S. cerevisiae)	3.5E-04	3
**1448291_at**	**Mmp9**	**matrix metallopeptidase 9**	**5.7E-04**	**3**
1418012_at	Sh3glb1	SH3-domain GRB2-like B1 (endophilin)	1.9E-04	3
1416492_at	Ccne1	cyclin E1	1.6E-04	3
1448127_at	Rrm1	ribonucleotide reductase M1	1.1E-05	3
1436472_at	Slfn9	schlafen 9	4.0E-04	3
**1418424_at**	**TNFaip6**	**tumor necrosis factor alpha induced protein 6**	**2.5E-04**	**3**
1419943_s_at	Ccnb1	cyclin B1	9.1E-04	3
1423690_s_at	Gpsm1	G-protein signalling modulator 1 (AGS3-like, C. elegans)	7.0E-04	3
1425295_at	Ear11	eosinophil-associated, ribonuclease A family, member 11	8.8E-04	3
1423774_a_at	Prc1	protein regulator of cytokinesis 1	4.8E-06	3
1454737_at	Dusp9	dual specificity phosphatase 9	1.4E-04	3
1438852_x_at	Mcm6	minichromosome maintenance deficient 6 (MIS5 homolog, S. pombe) (S. cerevisiae)	2.3E-04	3
1418133_at	Bcl3	B-cell leukemia/lymphoma 3	8.4E-04	3

1439269_x_at	Mcm7	minichromosome maintenance deficient 7 (S. cerevisiae)	3.7E-04	3
1435785_at	Ehd2	EH-domain containing 2	1.3E-04	3
1424501_at	Utp6	UTP6, small subunit (SSU) processome component, homolog (yeast)	2.6E-04	3
1452854_at	Sec63	SEC63-like (S. cerevisiae)	6.3E-05	3
1416961_at	Bub1b	budding uninhibited by benzimidazoles 1 homolog, beta (S. cerevisiae)	2.5E-04	3
1427183_at	Efemp1	epidermal growth factor-containing fibulin-like extracellular matrix protein 1	3.9E-04	3
1449513_at	Adam24	a disintegrin and metallopeptidase domain 24 (testase 1)	9.8E-04	3
1451782_a_at	Slc29a1	solute carrier family 29 (nucleoside transporters), member 1	6.9E-05	3
1424278_a_at	Birc5	baculoviral IAP repeat-containing 5	6.9E-04	3
1444459_at		Adult male urinary bladder cDNA, RIKEN full-length enriched library, clone:9530065A06 product:unclassifiable, full insert sequence	5.4E-04	3
1417856_at	Relb	avian reticuloendotheliosis viral (v-rel) oncogene related B	3.7E-04	3
1451989_a_at	Mapre2	microtubule-associated protein, RP/EB family, member 2	2.6E-06	3
1433807_at	6720463M24Rik	RIKEN cDNA 6720463M24 gene	3.1E-04	3
1439208_at	Chek1	checkpoint kinase 1 homolog (S. pombe)	6.2E-05	3
1435184_at	Npr3	natriuretic peptide receptor 3	8.5E-04	3
1417133_at	Pmp22	peripheral myelin protein	4.1E-04	3
1421052_a_at	Sms	spermine synthase	8.4E-04	3
1419938_s_at	Arhgef17	Rho guanine nucleotide exchange factor (GEF) 17	2.0E-04	3
1434475_at	Ppig	peptidyl-prolyl isomerase G (cyclophilin G)	3.5E-06	3
1427105_at	Cenpn	centromere protein N	2.1E-04	3
1424292_at	Depdc1a	DEP domain containing 1a	1.4E-04	3
1433813_at	Tmem48	transmembrane protein 48	6.0E-04	3
1460227_at	Timp1	tissue inhibitor of metalloproteinase 1	2.1E-04	3
1417534_at	Itgb5	integrin beta 5	4.2E-04	3
1448113_at	Stmn1	stathmin 1	2.4E-04	3
1442148_at	Psip1	PC4 and SFRS1 interacting protein 1	4.0E-04	3
1440478_at	LOC100047601	similar to DNA segment, Chr 10, ERATO Doi 438, expressed	4.1E-04	3
1420913_at	Slco2a1	solute carrier organic anion transporter family, member 2a1	1.0E-04	3
1449494_at	Rab3c	RAB3C, member RAS oncogene family	4.0E-04	3
1441559_at	LOC627626	similar to CG11212-PA	4.5E-04	3
1423666_s_at	Rpl5	ribosomal protein L5	1.7E-04	3
1446331_at	Ptgfr	prostaglandin F receptor	5.5E-04	3
1417533_a_at	Itgb5	integrin beta 5	3.9E-04	3
1448205_at	Ccnb1 /// Ccnb1-rs1	cyclin B1, related sequence 1 /// cyclin B1	8.4E-04	3
1417040_a_at	Bok	Bcl-2-related ovarian killer protein	2.4E-04	3
1435221_at		Adult male corpora quadrigemina cDNA, RIKEN full-length enriched library, clone:B230341P20 product:inferred: forkhead box P1, full insert sequence	3.6E-04	3
1423092_at	Incenp	inner centromere protein	1.8E-04	3
1439814_at		Transcribed locus	4.1E-04	3
1448899_s_at	Rad51ap1	RAD51 associated protein 1	4.7E-05	3
1455242_at	Foxp1	forkhead box P1	1.2E-04	3
1422814_at	Aspm	asp (abnormal spindle)-like, microcephaly associated (Drosophila)	9.6E-04	3
1449705_x_at	Mcm3	minichromosome maintenance deficient 3 (S. cerevisiae)	1.0E-05	3
1435162_at	Prkg2	protein kinase, cGMP-dependent, type II	3.5E-04	3
1425142_a_at	Hnrpd	heterogeneous nuclear ribonucleoprotein D	2.4E-04	3
