# Supplementary material for: The Mycoplasma hyorhinis p37 Protein Rapidly Induces Genes in Fibroblasts Associated with Inflammation and Cancer
Source: PLoS One. 2015 Oct 29;10(10):e0140753. doi: 10.1371/journal.pone.0140753 (PMC4626034; doi:10.1371/journal.pone.0140753)
Supplement: S7 Table — The dataset consists of 249 genes significantly downregulated by ≥ 3 fold with a p-value of ≤ 0.001. (PDF) [file pone.0140753.s015.pdf]

Affymetrix Probe Set ID	Gene Symbol	Gene Title	p-value	Fold Change (Down)
**1449382_at**	**Slc6a12**	**solute carrier family 6 (neurotransmitter transporter, betaine/GABA), member 12**	**1.5E-06**	**47**
**1438109_at**	**Clca5**	**chloride channel calcium activated 5**	**1.4E-04**	**32**
1434195_at	Prss35	protease, serine, 35	2.6E-05	27
1455477_s_at	Pdzk1ip1	PDZK1 interacting protein 1	3.7E-05	21
1451322_at	Cmbl	carboxymethylenebutenolidase-like (Pseudomonas)	2.6E-04	20
**1419378_a_at**	**Fxyd2**	**FXYD domain-containing ion transport regulator 2**	**2.4E-06**	**19**
1422478_a_at	Acss2	acyl-CoA synthetase short-chain family member 2	4.1E-07	18
1456601_x_at	Fxyd2	FXYD domain-containing ion transport regulator 2	7.1E-07	17
1422479_at	Acss2	acyl-CoA synthetase short-chain family member 2	6.8E-07	16
**1421153_at**	**Loxl4**	**lysyl oxidase-like 4**	**2.7E-05**	**16**
**1418925_at**	**Celsr1**	**cadherin EGF LAG seven-pass G-type receptor 1**	**3.4E-05**	**14**
1417689_a_at	Pdzk1ip1	PDZK1 interacting protein 1	1.2E-04	13
1418595_at	S3-12	plasma membrane associated protein, S3-12	4.6E-05	13
1443921_at	Ranbp3l	RAN binding protein 3-like	5.3E-04	13
1419379_x_at	Fxyd2	FXYD domain-containing ion transport regulator 2	1.4E-04	13
1439036_a_at	Atp1b1	ATPase, Na+/K+ transporting, beta 1 polypeptide	1.7E-04	12
1440700_a_at	Arhgef18	rho/rac guanine nucleotide exchange factor (GEF) 18	1.3E-04	12
1452388_at	Hspa1a	heat shock protein 1A	2.7E-05	12
1419872_at	Csf1r	colony stimulating factor 1 receptor	1.5E-06	12
1452418_at	Rik	RIKEN cDNA 1200016E24 gene	9.6E-04	12
1420558_at	Selp	selectin, platelet	4.8E-04	12
1416702_at	Serpini1	serine (or cysteine) peptidase inhibitor, clade I, member 1	2.1E-05	11
1456060_at	Maf	avian musculoaponeurotic fibrosarcoma (v-maf) AS42 oncogene homolog	4.5E-04	11
1418453_a_at	Atp1b1	ATPase, Na+/K+ transporting, beta 1 polypeptide	2.8E-05	11
1448831_at	Angpt2	angiopoietin 2	7.4E-05	11
1425784_a_at	Olfm1	olfactomedin 1	3.8E-06	11
1417150_at	Slc6a4	solute carrier family 6 (neurotransmitter transporter, serotonin), member 4	5.4E-05	11
1427919_at	Srpx2	sushi-repeat-containing protein, X-linked 2	3.4E-05	11
1455796_x_at	Olfm1	olfactomedin 1	1.1E-06	10
1448619_at	Dhcr7	7-dehydrocholesterol reductase	1.1E-04	10
1425124_at	Rnf183	ring finger protein 183	7.2E-04	10
1450682_at	Fabp6	fatty acid binding protein 6, ileal (gastrotropin)	3.9E-05	10
1419873_s_at	Csf1r	colony stimulating factor 1 receptor	1.0E-04	10
1440173_x_at	Selp	selectin, platelet	1.7E-04	10
1460336_at	Ppargc1a	peroxisome proliferative activated receptor, gamma, coactivator 1 alpha	1.2E-05	10
1449906_at	Selp	selectin, platelet	3.9E-04	9
1458385_at	Hspa4l	heat shock protein 4 like	7.5E-04	9
1420354_at	Cnnm1	cyclin M1	8.2E-05	9
1416831_at	Neu1	neuraminidase 1	5.6E-05	9

1427359_at	Jhdm1d	jumonji C domain-containing histone demethylase 1 homolog D (S. cerevisiae)	4.2E-05	9
1436948_a_at	6430550H21Rik	RIKEN cDNA 6430550H21 gene	1.7E-04	8
1415935_at	Smoc2	SPARC related modular calcium binding 2	1.1E-05	8
1443841_x_at	Uap1l1	UDP-N-acteylglucosamine pyrophosphorylase 1-like 1	2.2E-04	8
1456768_a_at	Mmrn2	multimerin 2	5.6E-04	8
1426516_a_at	Lpin1	lipin 1	3.9E-05	8
1448136_at	Enpp2	ectonucleotide pyrophosphatase/phosphodiesterase 2	7.1E-05	8
1416455_a_at	Cryab	crystallin, alpha B	2.0E-04	8
1452946_a_at	Rftn2	raftlin family member 2	1.9E-04	8
1456395_at	Ppargc1a	peroxisome proliferative activated receptor, gamma, coactivator 1 alpha	7.4E-05	7
1436188_a_at	Ndrg4	N-myc downstream regulated gene 4	1.4E-04	7
1453265_at	4930579C15Rik	RIKEN cDNA 4930579C15 gene	1.3E-04	7
1429676_at		Adult male stomach cDNA, RIKEN full-length enriched library, clone:2210408O09 product:unclassifiable, full insert sequence	9.6E-04	7
1436742_a_at	Gm1967	gene model 1967, (NCBI)	1.7E-05	7
1449010_at	Hspa4l	heat shock protein 4 like	1.4E-05	7
1448663_s_at	Mvd	mevalonate (diphospho) decarboxylase	1.0E-04	7
1418288_at	Lpin1	lipin 1	8.8E-05	7
1455707_at	Ranbp3l	RAN binding protein 3-like	3.7E-04	7
1415894_at	Enpp2	ectonucleotide pyrophosphatase/phosphodiesterase 2	5.4E-05	6
1460684_at	Tm7sf2	transmembrane 7 superfamily member 2	7.7E-05	6
1448421_s_at	Aspn	asporin	4.2E-04	6
1437751_at	Ppargc1a	peroxisome proliferative activated receptor, gamma, coactivator 1 alpha	9.4E-05	6
1439006_x_at	6430550H21Rik	RIKEN cDNA 6430550H21 gene	4.7E-05	6
1428074_at	Tmem158	transmembrane protein 158	6.0E-06	6
1424463_at	2210010L05Rik	RIKEN cDNA 2210010L05 gene	1.1E-04	6
1419704_at	Cyp3a41a /// LOC100041375	cytochrome P450, family 3, subfamily a, polypeptide 41A /// similar to cytochrome P450	1.8E-05	6
1422155_at	Hist2h3c2	histone cluster 2, H3c2	5.7E-05	6
1422916_at	Fgf21	fibroblast growth factor 21	6.0E-05	6
1426663_s_at	Slc45a3	solute carrier family 45, member 3	3.5E-04	6
1434099_at	Ppargc1a	Peroxisome proliferative activated receptor, gamma, coactivator 1 alpha	7.5E-05	6
1419684_at	Ccl8 /// LOC100048554	chemokine (C-C motif) ligand 8 /// similar to monocyte chemoattractant protein-2 (MCP-2)	1.4E-04	6
1443653_at			8.5E-04	6
1418818_at	Aqp5 /// LOC100046616	aquaporin 5 /// similar to aquaporin 5	8.6E-04	6
1422170_at	Slc5a3	solute carrier family 5 (inositol transporters), member 3	9.9E-05	6
1435849_at	6330417G02Rik	RIKEN cDNA 6330417G02 gene	8.9E-04	6
1417263_at	Ptgs2	prostaglandin-endoperoxide synthase 2	1.1E-04	6
1460033_at	C030002C11Rik	RIKEN cDNA C030002C11 gene	7.9E-04	6
1452117_a_at	Fyb	FYN binding protein	1.2E-04	6
1426615_s_at	Ndrg4	N-myc downstream regulated gene 4	2.8E-04	6
1443503_at	Ihpk3	inositol hexaphosphate kinase 3	8.1E-05	6
1434100_x_at	Ppargc1a	Peroxisome proliferative activated receptor,	1.1E-05	6

		gamma, coactivator 1 alpha		
1458562_at			7.0E-05	6
1418025_at	Bhlhb2	basic helix-loop-helix domain containing, class B2	6.0E-05	6
1435761_at	Stfa1	stefin A1 /// cDNA sequence BC100530	9.4E-04	5
1417303_at	Mvd	mevalonate (diphospho) decarboxylase	1.2E-04	5
1429888_a_at	Hspb2	heat shock protein 2	2.1E-05	5
1418253_a_at	Hspa4l	heat shock protein 4 like	7.0E-05	5
1438160_x_at	Slco4a1	solute carrier organic anion transporter family, member 4a1	1.4E-04	5
1449848_at	Gna14	guanine nucleotide binding protein, alpha 14	8.4E-04	5
1416069_at	Pfkp	phosphofructokinase, platelet	4.2E-05	5
1456319_at			1.0E-04	5
1420013_s_at	Lss	lanosterol synthase	2.6E-04	5
1426562_a_at	Olfm1	olfactomedin 1	1.3E-04	5
1439093_at			2.1E-04	5
1439518_at	Mmrn2	multimerin 2	2.0E-04	5
1435680_a_at	Dpp7	dipeptidylpeptidase 7	5.0E-06	5
1451486_at	Slc46a3	solute carrier family 46, member 3	2.9E-04	5
1437332_at		Adult male corpora quadrigemina cDNA, RIKEN full-length enriched library, clone:B230334A10 product:unclassifiable, full insert sequence	3.9E-04	5
1435867_at	ENSMUSG00000073143	predicted gene, ENSMUSG00000073143	1.0E-04	5
1426664_x_at	Slc45a3	solute carrier family 45, member 3	2.8E-06	5
1434046_at	AA467197	expressed sequence AA467197	2.0E-04	5
1438704_at	Trim63	tripartite motif-containing 63	7.4E-04	5
1428896_at	Pdgfrl	platelet-derived growth factor receptor-like	2.3E-05	5
1438211_s_at	Dbp	D site albumin promoter binding protein	9.1E-06	5
1423606_at	Postn	periostin, osteoblast specific factor	1.1E-04	5
1436842_at	B230380D07Rik	RIKEN cDNA B230380D07 gene	8.9E-05	5
1425113_x_at		Transcribed locus	9.6E-05	5
1418188_a_at	Malat1	Metastasis associated lung adenocarcinoma transcript 1 (non-coding RNA)	1.2E-04	5
1449363_at	Atf3	activating transcription factor 3	9.5E-06	5
1428379_at	Slc17a6	solute carrier family 17 (sodium-dependent inorganic phosphate cotransporter), member 6	5.2E-05	5
1429106_at	4921509J17Rik	RIKEN cDNA 4921509J17 gene	1.5E-05	5
1436480_at	Dpp7	dipeptidylpeptidase 7	1.1E-04	5
1430964_at	2310034O05Rik	RIKEN cDNA 2310034O05 gene	2.2E-04	5
1451382_at	Chac1	ChaC, cation transport regulator-like 1 (E. coli)	9.4E-05	5
1437123_at	Mmrn2	multimerin 2	5.5E-05	5
1428834_at	Dusp4	dual specificity phosphatase 4	6.1E-04	5
1422529_s_at	Casq2	calsequestrin 2	8.8E-05	5
1451457_at	Sc5d	sterol-C5-desaturase (fungal ERG3, delta-5-desaturase) homolog (S. cerevisae)	1.5E-04	5
1418219_at	Il15	interleukin 15	1.1E-04	5
1416046_a_at	Fuca2	fucosidase, alpha-L- 2, plasma	7.9E-05	5
1427126_at	Hspa1b	heat shock protein 1B	1.4E-04	5
1421594_a_at	Sytl2	synaptotagmin-like 2	4.6E-05	5
1439072_at	Slc1a3	solute carrier family 1 (glial high affinity glutamate transporter), member 3	9.5E-05	5
1440227_at	Slc5a3	solute carrier family 5 (inositol transporters), member 3	3.4E-04	5

1450922_a_at	Tgfb2	transforming growth factor, beta 2	9.5E-04	5
1436479_a_at	Dpp7	dipeptidylpeptidase 7	2.0E-04	5
1452132_at	Tlcd1	TLC domain containing 1	1.5E-05	5
1449702_at	Zfand2a	zinc finger, AN1-type domain 2A	1.0E-04	5
1416625_at	Serping1	serine (or cysteine) peptidase inhibitor, clade G, member 1	6.2E-05	5
1429236_at	Galntl2	UDP-N-acetyl-alpha-D-galactosamine:polypeptide N-acetylgalactosaminyltransferase-like 2	5.2E-04	5
1430619_a_at	Mvk	mevalonate kinase	6.1E-05	5
1429060_at			8.4E-04	5
1424464_s_at	2210010L05Rik	RIKEN cDNA 2210010L05 gene	4.6E-04	5
1433944_at	Hectd2	HECT domain containing 2	1.0E-03	5
1455065_x_at	Gnpda1	glucosamine-6-phosphate deaminase 1	1.8E-04	4
1418359_at	Wbscr27	Williams Beuren syndrome chromosome region 27 (human)	1.8E-04	4
1452583_s_at	Galm	galactose mutarotase	1.3E-04	4
1424140_at	Gale	galactose-4-epimerase, UDP	8.0E-05	4
1434191_at	A530016O06Rik	RIKEN cDNA A530016O06 gene	2.6E-04	4
1450745_at	C1galt1	core 1 synthase, glycoprotein-N-acetylgalactosamine 3-beta-galactosyltransferase, 1	1.7E-05	4
1448596_at	Slc6a8	solute carrier family 6 (neurotransmitter transporter, creatine), member 8	5.3E-04	4
1426616_at	Tlcd1	TLC domain containing 1	2.9E-05	4
1440929_at			9.1E-04	4
1427893_a_at	Pmvk	phosphomevalonate kinase	8.5E-04	4
1419663_at	Ogn	osteoglycin	8.8E-05	4
1432976_at	2310038E17Rik	RIKEN cDNA 2310038E17 gene	2.9E-04	4
1423890_x_at	Atp1b1	ATPase, Na+/K+ transporting, beta 1 polypeptide	7.6E-05	4
1448443_at	Serpini1	serine (or cysteine) peptidase inhibitor, clade I, member 1	1.8E-05	4
1415941_s_at	Zfand2a	zinc finger, AN1-type domain 2A	6.2E-04	4
1427534_at	4930535I16Rik	RIKEN cDNA 4930535I16 gene	4.0E-05	4
1436325_at	Rora	RAR-related orphan receptor alpha	6.0E-04	4
1421571_a_at	Ly6c2	lymphocyte antigen 6 complex, locus C1	4.2E-05	4
1441281_s_at	Ninj1	ninjurin 1	1.3E-05	4
1456611_at	D430015B01Rik	RIKEN cDNA D430015B01 gene	6.5E-04	4
1455269_a_at	Coro1a	coronin, actin binding protein 1A	1.5E-04	4
1441971_at		Transcribed locus	1.9E-04	4
1460122_at	Tmem41b	transmembrane protein 41B	2.6E-06	4
1450378_at	Tapbp	TAP binding protein	2.4E-04	4
1450134_at	Loxl4	lysyl oxidase-like 4	1.8E-04	4
1427287_s_at	Itpr2	inositol 1,4,5-triphosphate receptor 2	3.5E-05	4
1424834_s_at	Itpr2	inositol 1,4,5-triphosphate receptor 2	1.8E-04	4
1434369_a_at	Cryab	crystallin, alpha B	1.4E-04	4
1417664_a_at	Ndrg3	N-myc downstream regulated gene 3	8.5E-05	4
1430307_a_at	Mod1	malic enzyme, supernatant	2.1E-05	4
1456590_x_at	Akr1b3	aldo-keto reductase family 1, member B3 (aldose reductase)	5.5E-05	4
1420062_at			2.9E-04	4
1452452_at		Intracisternal A-particle-related retroviral elements and envelope pseudogene	2.6E-04	4

1431046_at	Ppfia3	protein tyrosine phosphatase, receptor type, f polypeptide (PTPRF), interacting protein (liprin), alpha 3	2.8E-04	4
1449954_at	Hyal1 /// Nat6	hyaluronoglucosaminidase 1 /// N-acetyltransferase 6	2.5E-04	4
1452646_at	Trp53inp2	transformation related protein 53 inducible nuclear protein 2	1.3E-04	4
1416114_at	Sparcl1	SPARC-like 1 (mast9, hevin)	1.5E-04	4
1427127_x_at	Hspa1b	heat shock protein 1B	9.7E-05	4
1440282_at	Tulp4	tubby like protein 4	4.2E-04	4
1448417_at	Ninj1	ninjurin 1	1.1E-05	4
1440397_at	Cacna2d1	calcium channel, voltage-dependent, alpha2/delta subunit 1	8.7E-04	4
1416632_at	Mod1	malic enzyme, supernatant	5.3E-04	4
1424167_a_at	Pmm1	phosphomannomutase 1	8.2E-05	4
1445694_at		Transcribed locus	1.5E-04	4
1438426_at	Tmem58	transmembrane protein 58	3.1E-04	4
1431780_at	1700021K14Rik	RIKEN cDNA 1700021K14 gene	1.6E-05	4
1426936_at	BC005512 /// EG641366 /// LOC629242	cDNA sequence BC005512 /// hypothetical protein LOC629242 /// predicted gene, EG641366	6.5E-04	4
1424172_at	Hagh	hydroxyacyl glutathione hydrolase	6.4E-05	4
1417110_at	Man1a	mannosidase 1, alpha	6.5E-05	4
1436841_at	B230380D07Rik	RIKEN cDNA B230380D07 gene	1.2E-06	4
1448481_at	Neu1	neuraminidase 1	2.3E-04	4
1416274_at	Ctns	cystinosis, nephropathic	5.8E-05	4
1447774_x_at	5730469M10Rik	RIKEN cDNA 5730469M10 gene	2.1E-04	4
1437519_x_at	Hagh /// LOC100044022	hydroxyacyl glutathione hydrolase /// similar to Hydroxyacyl glutathione hydrolase	1.8E-04	4
1453011_at	Bdh2	3-hydroxybutyrate dehydrogenase, type 2	4.3E-04	4
1422433_s_at	Idh1	isocitrate dehydrogenase 1 (NADP+), soluble	3.9E-04	4
1436528_at	Kazald1	Kazal-type serine peptidase inhibitor domain 1	4.1E-04	4
1457102_at	A030001D16Rik	RIKEN cDNA A030001D16 gene	6.1E-05	4
1424443_at	Hdgfrp3 /// Tm6sf1	hepatoma-derived growth factor, related protein 3 /// transmembrane 6 superfamily member 1	6.3E-04	4
1429239_a_at	Stard4	StAR-related lipid transfer (START) domain containing 4	5.5E-04	4
1417109_at	Tinagl	tubulointerstitial nephritis antigen-like	6.4E-04	4
1458264_at	AW046200	Expressed sequence AW046200	1.8E-04	4
1448830_at	Dusp1	dual specificity phosphatase 1	8.4E-05	4
1424171_a_at	Hagh	hydroxyacyl glutathione hydrolase	1.9E-04	4
1417871_at	Hsd17b7	hydroxysteroid (17-beta) dehydrogenase 7	9.3E-05	4
1449869_at	Vpreb1	pre-B lymphocyte gene 1	7.8E-05	4
1417049_at	Rhd	Rh blood group, D antigen	4.9E-04	4
1450678_at	Itgb2	integrin beta 2	2.1E-04	4
1421061_at	Guca1a	guanylate cyclase activator 1a (retina)	4.1E-05	4
1452716_at	5730469M10Rik	RIKEN cDNA 5730469M10 gene	5.5E-04	4
1418601_at	Aldh1a7	aldehyde dehydrogenase family 1, subfamily A7	4.3E-05	4
1447252_s_at	Mep1a	meprin 1 alpha	2.8E-04	4
1454797_at	Tmem55b	transmembrane protein 55b	2.0E-05	4
1420693_at	Myom1	myomesin 1	1.1E-04	4
1427285_s_at	Malat1	metastasis associated lung adenocarcinoma transcript 1 (non-coding RNA)	6.4E-04	4

1416926_at	Trp53inp1	transformation related protein 53 inducible nuclear protein 1	2.6E-04	4
1448183_a_at	Hif1a	hypoxia inducible factor 1, alpha subunit	4.2E-04	4
1423250_a_at	Tgfb2	transforming growth factor, beta 2	5.4E-04	4
1415940_at	Zfand2a	zinc finger, AN1-type domain 2A	2.6E-04	4
1445580_at			3.9E-04	4
1429777_at	Dnajb6	DnaJ (Hsp40) homolog, subfamily B, member 6	1.3E-04	4
1441288_at		Transcribed locus	6.1E-04	4
1435124_at	EG328644	predicted gene, EG328644	7.4E-04	4
1419132_at	TLR2	toll-like receptor 2	1.0E-04	4
1422924_at	TNFsf9	tumor necrosis factor (ligand) superfamily, member 9	2.7E-04	4
1449408_at	Jam2	junction adhesion molecule 2	7.6E-04	4
1426886_at	Cln5	ceroid-lipofuscinosis, neuronal 5	6.2E-05	4
1427546_at	Abca8b	ATP-binding cassette, sub-family A (ABC1), member 8b	1.0E-04	4
1430780_a_at	Pmm1	phosphomannomutase 1	1.3E-04	4
1447791_s_at	Gna14	guanine nucleotide binding protein, alpha 14	3.7E-04	4
1420784_at	Scn11a	sodium channel, voltage-gated, type XI, alpha	7.7E-04	4
1416246_a_at	Coro1a	coronin, actin binding protein 1A	2.5E-05	4
1438928_x_at	Ninj1	ninjurin 1	1.0E-04	4
1419124_at	2210010L05Rik	RIKEN cDNA 2210010L05 gene	1.4E-04	4
1436634_at	Robo3	roundabout homolog 3 (Drosophila)	6.5E-04	4
1417382_at	Entpd5	ectonucleoside triphosphate diphosphohydrolase 5	6.0E-05	4
1453008_at	2300002D11Rik	RIKEN cDNA 2300002D11 gene	2.7E-04	4
1418715_at	Pank1	pantothenate kinase 1	3.3E-04	4
1437642_at	Hrbl	HIV-1 Rev binding protein-like	9.9E-06	4
1426908_at	Galnt7	UDP-N-acetyl-alpha-D-galactosamine: polypeptide N-acetylgalactosaminyltransferase 7	4.4E-04	4
1454757_s_at	D12Ertd647e	DNA segment, Chr 12, ERATO Doi 647, expressed	3.3E-05	4
1421812_at	Tapbp	TAP binding protein	2.0E-06	4
1452837_at	Lpin2	lipin 2	4.2E-04	4
1460346_at	Arsa	arylsulfatase A	2.5E-04	4
1438266_at	Adamtsl5	ADAMTS-like 5	5.6E-04	4
1441952_x_at	Lynx1	Ly6/neurotoxin 1	5.0E-04	4
1454161_s_at	0610007P14Rik	RIKEN cDNA 0610007P14 gene	5.4E-05	4
1416968_a_at	Hsd3b7	hydroxy-delta-5-steroid dehydrogenase, 3 beta- and steroid delta-isomerase 7	8.3E-04	3
1452014_a_at	Igf1	insulin-like growth factor 1	5.8E-04	3
1419665_a_at	Nupr1	nuclear protein 1	4.3E-04	3
1453054_at	Scamp1	secretory carrier membrane protein 1	1.4E-04	3
1455717_s_at	Daam2	dishevelled associated activator of morphogenesis 2	1.6E-04	3
1418367_x_at	Hist1h2ad	histone cluster 2, H2aa1	6.7E-05	3
1415824_at	Scd2	stearoyl-Coenzyme A desaturase 2	3.9E-04	3
1420385_at	Gna14	guanine nucleotide binding protein, alpha 14	2.8E-04	3
1416262_at	Tmem19	transmembrane protein 19	2.9E-04	3
1454699_at	LOC100047324 /// Sesn1	sestrin 1 /// similar to Sesn1 protein	8.0E-05	3
1428025_s_at	Pitpnc1	phosphatidylinositol transfer protein, cytoplasmic 1	4.1E-04	3
1424940_s_at	BC022687	cDNA sequence BC022687	2.5E-04	3

1428190_at	Slc25a1	solute carrier family 25 (mitochondrial carrier, citrate transporter), member 1	1.2E-04	3
1452789_at	Snn	stannin	3.0E-05	3
1453080_at	Apol3	apolipoprotein L 3	4.1E-04	3
1434849_at	Tspyl2	TSPY-like 2	4.0E-04	3
1452318_a_at	Hspa1b	heat shock protein 1B	3.3E-04	3
1415865_s_at	Bpgm	2,3-bisphosphoglycerate mutase	5.3E-04	3
1454706_at	Uvrag	UV radiation resistance associated gene	1.5E-04	3
1424034_at	Rora	RAR-related orphan receptor alpha	4.9E-06	3
1418536_at	H2-Q6	histocompatibility 2, Q region locus 7	2.9E-07	3
1452290_at	Tmem106b	transmembrane protein 106B	1.2E-05	3
1459871_x_at	Mar-02	membrane-associated ring finger (C3HC4) 2	8.9E-05	3
1417283_at	Lynx1	Ly6/neurotoxin 1	3.3E-04	3
1436890_at	Uap1l1	UDP-N-acteylglucosamine pyrophosphorylase 1-like 1	2.0E-04	3
1435135_at	Aadacl1	arylacetamide deacetylase-like 1	4.4E-04	3
1431128_at	Tmem170	transmembrane protein 170	1.8E-04	3
1451533_at	BC022687	cDNA sequence BC022687	4.2E-04	3
1460022_at		Adult male epididymis cDNA, RIKEN full-length enriched library, clone:9230106K18 product:unclassifiable, full insert sequence	1.2E-04	3
1428352_at	Arrdc2	arrestin domain containing 2	3.4E-04	3
1421856_at	S100a3	S100 calcium binding protein A3	5.9E-04	3
1419100_at	Serpina3n	serine (or cysteine) peptidase inhibitor, clade A, member 3N	9.7E-04	3
1418435_at	Mkrn1	makorin, ring finger protein, 1	2.9E-04	3
1433711_s_at	LOC100047324 /// Sesn1	sestrin 1 /// similar to Sesn1 protein	4.3E-06	3
1437597_at		Transcribed locus	7.1E-05	3
1448163_at	Gnpda1	glucosamine-6-phosphate deaminase 1	4.8E-04	3
1435071_at	Zfyve1	zinc finger, FYVE domain containing 1	5.6E-04	3
1425281_a_at	Tsc22d3	TSC22 domain family 3	5.0E-05	3
1456721_at	Thsd7a	thrombospondin, type I, domain containing 7A	6.9E-04	3
1428895_at	Rftn2	raftlin family member 2	5.4E-04	3
1429841_at	Megf10	multiple EGF-like-domains 10	3.3E-04	3
1435902_at	Nudt18	nudix (nucleoside diphosphate linked moiety X)-type motif 18	6.8E-05	3
1448148_at	Grn	granulin	8.4E-04	3
1428586_at	Tmem41b	transmembrane protein 41B	6.3E-04	3
1442947_x_at		13 days embryo heart cDNA, RIKEN full-length enriched library, clone:D330042P15 product:unclassifiable, full insert sequence	8.6E-04	3
1422064_a_at	Zbtb20	zinc finger and BTB domain containing 20	9.4E-04	3
1415864_at	Bpgm	2,3-bisphosphoglycerate mutase	5.8E-04	3
1450798_at	Tnxb	tenascin XB	8.1E-05	3
1423086_at	Npc1	Niemann Pick type C1	4.0E-05	3
1453102_at	Flrt3	fibronectin leucine rich transmembrane protein 3	3.3E-04	3
1422670_at	Rnd2	Rho family GTPase 2	2.6E-04	3
1433588_at	D6Wsu116e	DNA segment, Chr 6, Wayne State University 116, expressed	3.9E-05	3
1422038_a_at	TNFrsf22	tumor necrosis factor receptor superfamily, member 22	1.5E-05	3

1438931_s_at	LOC100047324 /// Sesn1	sestrin 1 /// similar to Sesn1 protein	4.5E-04	3
1437261_at	2900024O10Rik	RIKEN cDNA 2900024O10 gene	1.3E-04	3
1437704_at	2900024O10Rik	RIKEN cDNA 2900024O10 gene	1.6E-04	3
1423141_at	Lipa	lysosomal acid lipase A	2.1E-04	3
1426851_a_at	Nov	nephroblastoma overexpressed gene	5.6E-05	3
1419030_at	Ero1l	ERO1-like (S. cerevisiae)	1.3E-04	3
1431417_at	Jam2	junction adhesion molecule 2	4.6E-04	3
1424785_at	Angptl6	angiopoietin-like 6	1.4E-04	3
1441649_at		0 day neonate lung cDNA, RIKEN full-length enriched library, clone:E030001E08 product:unclassifiable, full insert sequence	8.0E-04	3
1445882_at	Cd300lb	CD300 antigen like family member B	6.7E-06	3
1432103_a_at	Sh3gl3	SH3-domain GRB2-like 3	4.0E-04	3
1431769_at	2210406O10Rik	RIKEN cDNA 2210406O10 gene	1.1E-04	3
1456744_x_at	Flcn	folliculin	5.5E-04	3
1450626_at	Manba	mannosidase, beta A, lysosomal	7.5E-04	3
1442140_at	Tnn	tenascin N	5.7E-04	3
1419666_x_at	Nupr1	nuclear protein 1	6.0E-04	3
1455011_at	Stard4	StAR-related lipid transfer (START) domain containing 4	2.3E-04	3
1424951_at	Baiap2l1	BAI1-associated protein 2-like 1	7.6E-05	3
1423593_a_at	Csf1r	colony stimulating factor 1 receptor	5.4E-04	3
1423662_at	Atp6ap2	ATPase, H+ transporting, lysosomal accessory protein 2	4.3E-04	3
1426315_a_at	6330416G13Rik	RIKEN cDNA 6330416G13 gene	2.6E-05	3
1438579_at	Utp14b	UTP14, U3 small nucleolar ribonucleoprotein, homolog B (yeast)	2.4E-04	3
1452956_a_at	D12Ertd647e	DNA segment, Chr 12, ERATO Doi 647, expressed	2.3E-04	3
1442893_at			4.7E-05	3
1452232_at	Galnt7	UDP-N-acetyl-alpha-D-galactosamine: polypeptide N-acetylgalactosaminyltransferase 7	6.8E-04	3
1418129_at	Dhcr24	24-dehydrocholesterol reductase	3.7E-04	3
1448696_at	Heph	hephaestin	3.4E-04	3
1417551_at	Cln3	ceroid lipofuscinosis, neuronal 3, juvenile (Batten, Spielmeyer-Vogt disease)	7.0E-04	3
1428195_at	4631427C17Rik	RIKEN cDNA 4631427C17 gene	1.9E-05	3
1425235_s_at	Col20a1	collagen, type XX, alpha 1	8.2E-04	3
1455820_x_at	Scarb1	scavenger receptor class B, member 1	9.2E-04	3
1428334_at	Ostm1	osteopetrosis associated transmembrane protein 1	1.4E-04	3
1435357_at	D4Wsu53e	DNA segment, Chr 4, Wayne State University 53, expressed	5.8E-04	3
1448200_at	Tcn2	transcobalamin 2	2.2E-04	3
1450708_at	Scg2	secretogranin II	1.2E-04	3
1423585_at	Igfbp7	insulin-like growth factor binding protein 7	5.4E-04	3
1426708_at	Antxr2	anthrax toxin receptor 2	8.4E-05	3
1441481_at	Mfap3l	microfibrillar-associated protein 3-like	1.6E-04	3
1435484_at	BF642829	expressed sequence BF642829	5.7E-05	3
1416830_at	0610031J06Rik	RIKEN cDNA 0610031J06 gene	7.7E-04	3
1433074_at	4931412I15Rik	RIKEN cDNA 4931412I15 gene	2.5E-04	3
1456056_a_at	D6Wsu116e	DNA segment, Chr 6, Wayne State University 116, expressed	4.5E-05	3

1433034_at	2310007H11Rik	RIKEN cDNA 2310007H11 gene	4.6E-05	3
1451799_at	Ccdc25	coiled-coil domain containing 25	7.7E-04	3
1450919_at	Mpp1	membrane protein, palmitoylated	2.1E-05	3
1419688_at	Gpc6	glypican 6	5.8E-04	3
1420833_at	Vamp2	vesicle-associated membrane protein 2	3.1E-04	3
1443882_at		Transcribed locus	2.8E-05	3
1437044_a_at	Gba	glucosidase, beta, acid	4.8E-04	3
1438167_x_at	Flcn	Folliculin	1.9E-04	3
1425140_at	Lactb2	lactamase, beta 2	1.9E-04	3
1456482_at	Pik3r3	phosphatidylinositol 3 kinase, regulatory subunit, polypeptide 3 (p55)	9.0E-05	3
1419006_s_at	Peli2	pellino 2	4.4E-04	3
1452271_at	Xpr1	xenotropic and polytropic retrovirus receptor 1	2.5E-04	3
1438415_s_at	Yipf2	Yip1 domain family, member 2	6.3E-04	3
1451152_a_at	Atp1b1	ATPase, Na+/K+ transporting, beta 1 polypeptide	4.4E-05	3
1448319_at	Akr1b3	aldo-keto reductase family 1, member B3 (aldose reductase)	4.7E-05	3
1416265_at	Capn10	calpain 10	7.9E-05	3
